# Maternal Melatonin Therapy Rescues Prenatal Dexamethasone and Postnatal High-Fat Diet Induced Programmed Hypertension in Male Rat Offspring

**DOI:** 10.3389/fphys.2015.00377

**Published:** 2015-12-11

**Authors:** You-Lin Tain, Jiunn-Ming Sheen, Hong-Ren Yu, Chih-Cheng Chen, Mao-Meng Tiao, Chien-Ning Hsu, Yu-Ju Lin, Kuang-Che Kuo, Li-Tung Huang

**Affiliations:** ^1^Department of Pediatrics, Kaohsiung Chang Gung Memorial Hospital and Chang Gung University, College of MedicineKaohsiung, Taiwan; ^2^Center for Translational Research in Biomedical Sciences, Kaohsiung Chang Gung Memorial Hospital and Chang Gung University, College of MedicineKaohsiung, Taiwan; ^3^Department of Pharmacy, Kaohsiung Chang Gung Memorial HospitalKaohsiung, Taiwan; ^4^School of Pharmacy, Kaohsiung Medical UniversityKaohsiung, Taiwan; ^5^Department of Obstetrics and Gynecology, Kaohsiung Chang Gung Memorial Hospital and Chang Gung University, College of MedicineKaohsiung, Taiwan; ^6^Department of Traditional Chinese Medicine, Chang Gung UniversityLinkou, Taiwan

**Keywords:** fat, glucocorticoid, hypertension, melatonin, next generation sequencing, renin-angiotensin system

## Abstract

Prenatal dexamethasone (DEX) exposure and high-fat (HF) intake are linked to hypertension. We examined whether maternal melatonin therapy prevents programmed hypertension synergistically induced by prenatal DEX plus postnatal HF in adult offspring. We also examined whether DEX and melatonin causes renal programming using next-generation RNA sequencing (NGS) technology. Pregnant Sprague-Dawley rats received intraperitoneal dexamethasone (0.1 mg/kg) or vehicle from gestational day 16 to 22. In the melatonin-treatment groups (M), rats received 0.01% melatonin in drinking water during their entire pregnancy and lactation. Male offspring were assigned to five groups: control, DEX, HF, DEX+HF, and DEX+HF+M. Male offspring in the HF group were fed a HF diet from weaning to 4 months of age. Prenatal DEX and postnatal HF diet synergistically induced programmed hypertension in adult offspring, which melatonin prevented. Maternal melatonin treatment modified over 3000 renal transcripts in the developing offspring kidney. Our NGS data indicate that PPAR signaling and fatty acid metabolism are two significantly regulated pathways. In addition, maternal melatonin therapy elicits longstanding alterations on renal programming, including regulation of the melatonin signaling pathway and upregulation of *Agtr1b* and *Mas1* expression in the renin-angiotensin system (RAS), to protect male offspring against programmed hypertension. Postnatal HF aggravates prenatal DEX induced programmed hypertension in adult offspring, which melatonin prevented. The protective effects of melatonin on programmed hypertension is associated with regulation of the RAS and melatonin receptors. The long-term effects of maternal melatonin therapy on renal transcriptome require further clarification.

## Introduction

Melatonin (N-acetyl-5-methoxytryptamine) is an endogenous indoleamine with pleiotropic bioactivities, including antioxidant and anti-inflammatory properties, regulation of the circadian rhythm and energy balance, control of reproduction, and epigenetic regulation (Chen et al., 2013; Galano et al., 2013; Cipolla-Neto et al., 2014; Tain et al., 2014b). Notably melatonin deficiency causes hypertension whereas melatonin has been used to treat hypertension in different experimental animal models (Simko and Paulis, 2007).

Hypertension can originate from early life by a process referred to as “developmental programming” (Ritz et al., 2011). We previously reported that melatonin therapy during pregnancy and lactation has long-term effect on adult offspring to prevent the development of hypertension in a variety of programming models, including maternal caloric restriction, prenatal, or neonatal dexamethasone (DEX) exposure, and maternal high-fructose intake (Wu et al., 2014; Tain et al., 2014a,c,d). Even though several organs are involved in blood pressure (BP) control, the developing kidney is particularly vulnerable to the insults of programming in early life. Accordingly, renal programming has been considered as a driving mechanism of programmed hypertension (Paixão and Alexander, 2013).

Glucocorticoids are necessary for development and maturation of the fetal organs. However, prenatal glucocorticoid excess programs several adverse outcomes in adult offspring, including hypertension (Moisiadis and Matthews, 2014). Our previous research has shown that prenatal DEX exposure induces programmed hypertension in adult offspring, which can be protected by maternal melatonin treatment (Tain et al., 2014a). Similar to melatonin, glucocorticoids plays a crucial role in BP control, energy balance, and epigenetic regulation (Nieuwenhuizen and Rutters, 2008; Moisiadis and Matthews, 2014). While melatonin is beneficial to reserving the adverse programming effects associated with compromised pregnancies (Chen et al., 2013), its effects on renal programming in the developing kidney remain unclear. Therefore, we first employed the whole-genome RNA next-generation sequencing (NGS) to analyze renal transcriptome in the 1-week-old kidneys from prenatal DEX, melatonin, and DEX + melatonin exposed male offspring, to capture candidate genes and pathways responsible for primary programmed changes.

Next, given that postnatal insults can act as a “second hit” to deteriorate earlier programming induced by a first hit (e.g., prenatal DEX), that fatty acid metabolism pathway was significantly altered in response to both DEX and melatonin from our NGS data, and that high-fat diets closely link to the development of hypertension (Damjanovic and Barton, 2008), we hypothesized that prenatal DEX enhances offspring vulnerability to postnatal HF-induced programmed hypertension and that melatonin can protect the adult offspring against the two-hit insults. The study was therefore designed to address two specific questions: First, to examine renal transcriptome at 1-week-old male offspring exposed to prenatal DEX and/or melatonin; Second, to test if maternal melatonin therapy can prevent programmed hypertension in the adult offspring exposed to prenatal DEX plus postnatal high-fat diet and to explore the underlying mechanisms.

## Material and methods

### Animals and experimental design

The protocol was approved by the Institutional Animal Care and Use Committee of the Kaohsiung Chang Gung Memorial Hospital. This experiment was performed under the Guidelines for Animal Experiments of Chang Gung Memorial Hospital and Chang Gung University. Virgin Sprague-Dawley (SD) rats (12–16 weeks old) were obtained from the BioLASCO Taiwan Co., Ltd. (Taipei, Taiwan). Male SD rats were caged with individual females until mating was confirmed by observation of a vaginal plug. All animals were housed in a room maintained at 22 ± 1°C with 12-h light/dark cycles (dark period from 1900 to 0700) in a facility accredited by the Association for Assessment and Accreditation of Laboratory Animal Care International.

The aim of the first series of experiments was to analyze renal transcriptome in 1-week-old offspring kidney. To construct a prenatal DEX exposure model, dexamethasone (0.1 mg/kg body weight [BW]) or vehicle was intraperitoneally administered to pregnant SD rats from gestational day 16 to 22 (Tain et al., 2014a). After birth, the subjects came from litters of eight pups to standardize the received quantity of milk and maternal pup care. Each litter was left with the mother until weaning; pups were not weighed at birth to prevent maternal rejection. Because hypertension occurs at a higher rate and at an earlier age in males than females (Reckelhoff, 2001), only male offspring was selected from each litter and used in subsequent experiments. Male offspring were assigned to four groups: control, DEX, control + melatonin, and DEX + melatonin. Melatonin-treated pregnant rats received 0.01% melatonin in drinking water during the entire pregnancy and lactation. The dose of melatonin used here was based on our previous study (Tain et al., 2014a). Melatonin was prepared two times a week by dissolving 10 mg of melatonin in 1 mL of 100% ethanol. This solution was then diluted with water to a final concentration of 0.01%. Water bottles were wrapped with aluminum foil to protect from light. Male offspring was sacrificed at 1 week of age.

Our second series of experiments assessed the effects of melatonin in a prenatal DEX plus postnatal high-fat (HF) diet model. Five groups were studied: control, DEX, HF, DEX+HF, and DEX+HF+M. DEX group was conducted as described earlier. Male offspring rats in HF group received high-fat diet (58% high-fat diet, Research Diet, D12331) from weaning to 4 months of age. Melatonin-treated pregnant rats received 0.01% melatonin in drinking water during the entire pregnancy and lactation.

BP was measured in conscious rats by an indirect tail-cuff method (BP-2000, Visitech Systems, Inc., Apex, NC, USA) (Tain et al., 2014a). One week prior to the experiment, rats were adapted to restraint and tail-cuff inflation. Their tails were passed through tail cuffs and secured in place with tape. Following a 10-min warm-up period, 10 preliminary cycles of tail-cuff inflation were performed to allow the rats to adjust to the inflating cuff. The BP measurements were taken between 1300 and 1700 each day. A total of five cycles were recorded at each time point. Average of values from three stable measurements was taken. At 16 weeks of age, male offspring were sacrificed in the early light phase of the light-dark cycle. The midline of the abdomen was opened, and the intestines were displaced laterally to allow visualization of the aorta. The aorta was dissected from the adjacent vena cava, connective tissue, and fat. The aorta was cannulated with a 20- to 23-gauge butterfly, heparinized blood samples were collected, the vena cava was cut, and PBS was perfused until the kidneys were blanched. Kidneys were harvested after perfusion, decapsulated, divided into cortex and medulla, and stored at −80°C for further analysis. Renal melatonin level was measured using an ELISA kit (MyBioSource, San Diego, CA, USA), according to the manufacturer's protocol. In short, 100 mg kidney cortex was homogenized in 500 μL PBS and protein concentration was determined by Bradford assay (Bio-Rad, Hercules, CA, USA). After two freeze-thaw cycles were performed, tissue homogenate samples were centrifuged. The supernatant was assayed. Duplicate determinations in 100 μL of homogenate samples were made and the average of two measurements was used in subsequent statistical analysis of the data. The melatonin level was quantified spectrophotometrically at 450 nm. The results were expressed as pg melatonin per mg of protein.

### Next-generation sequencing and analysis

Kidney cortex (*n* = 3/group) isolated from first series of experiment was used for whole-genome RNA next-generation sequencing (NGS; Welgene Biotech Co., Ltd., Taipei, Taiwan). Purified RNA was quantified at 260 nm (OD_600_) by using a ND-1000 spectrophotometer (Nanodrop Technology, Wilmington, DE, USA) and analyzed using a Bioanalyzer 2100 (Agilent Technology) with RNA 6000 LabChip kit (Agilent Technologies). All procedures were performed according to the Illumina protocol. Using the TruSeq SBS Kit, the sequence was determined by sequencing-by-synthesis technology. Raw sequences were obtained using the Illumina GA Pipeline software CASAVA v1.8, which was expected to generate 10 million reads per sample. Quantification for gene expression was calculated as reads per kilobase of exon per million mapped reads (RPKM) (Mortazavi et al., 2008). For differential expression analysis, CummeRbund (Illumina Inc.) was employed to perform statistical analyses of gene expression profiles. The cuffdiff tool from the cufflinks package (Trapnell et al., 2013) was run to calculate expression changes and associated *q*-values (false discovery rate adjusted *p*-values) for each gene between control and DEX, control and M, as well as control and DEX+M groups. The output files of cuffdiff were further annotated by adding gene functional descriptions and Gene Ontology (GO) classifications. GO term enrichment and fold enrichment or depletion for gene lists of significantly up- and downregulated genes in kidney were determined. GO analysis for significant genes was performed using Kyoto Encyclopedia of Genes and Genomes (KEGG) and NIH DAVID Bioinformatics Resources 6.7 (Dennis et al., 2003) to identify regulated biological themes.

### Quantitative real-time PCR analysis

RNA was extracted as previously described procedures (Tain et al., 2014a). Two-step quantitative real-time PCR (qPCR) was conducted using Quantitect SYBR Green PCR Reagents (Qiagen, Valencia, CA) on a LightCycler 480 Real-Time PCR System (Roche Co., Germany). Several components of renin-angiotensin system (RAS) analyzed in this study included renin (*Ren*), (pro)renin receptor (*Atp6ap2*), angiotensinogen (Agt), angiotensin converting enzyme-1 and -2 (*Ace and Ace2*), angiotensin II type 1 & 2 receptor (*Agtr1a* and *Agtr1b*), and angiotensin (1–7) receptor *Mas1*. *R18S* was used as a housekeeping gene in all analyses as it did not change its threshold cycle (C_T_) in control and the prenatal DEX model (Tain et al., 2014a). Primers were designed using GeneTool Software (Biotools, Edmonton, Alberta, Canada) (Table S1). All samples were run in duplicate. For the relative quantification of gene expression, the comparative C_T_ method was employed. The averaged C_T_ was subtracted from the corresponding averaged *r18S* value for each sample, resulting in ΔC_T_. ΔΔC_T_ was achieved by subtracting the average control ΔC_T_ value from the average experimental ΔC_T_. The fold-increase was established by calculating 2^−ΔΔCT^ for experimental vs. reference samples.

### Western blot

Western blot analysis was performed as we published previously (Tain et al., 2014a,d; Wu et al., 2014). Three melatonin receptors, including melatonin receptor-1 (MT1), and -2 (MT2), and RORα, were analyzed. We used the following antibodies: a goat anti-rat MT1 antibody (1:1000, overnight incubation; Santa Cruz Biotechnology, Santa Cruz, CA, USA); a rabbit anti-rat MT2 antibody (1:1000, overnight incubation; Biorbyt, AllBio Science Inc., Taichung, Taiwan); and a rabbit anti-rat RORα antibody (1:2000, overnight incubation; Proteintech Group, Inc., Chicago, IL, USA). Bands of interest were visualized using SuperSignal West Pico reagent (Pierce; Rockford, IL, USA) and quantified by densitometry as integrated optical density (IOD), factored for Ponceau S red (PonS) staining to correct for any variations in total protein loading and for an internal standard. The protein abundance was represented as IOD/PonS.

### Statistics

Normally distributed data were given as mean ± S.E.M. For most parameters, statistical analysis was performed using One-way ANOVA with Tukey's *post-hoc* test for multiple comparisons. BP was analyzed by Two-way repeated-measures ANOVA and Tukey's *post-hoc* test. A *P* < 0.05 was considered statistically significant. All analyses were performed using the Statistical Package for the Social Sciences software (SPSS; IBM, Armonk, NY, USA).

## Results

**Figure 1:**
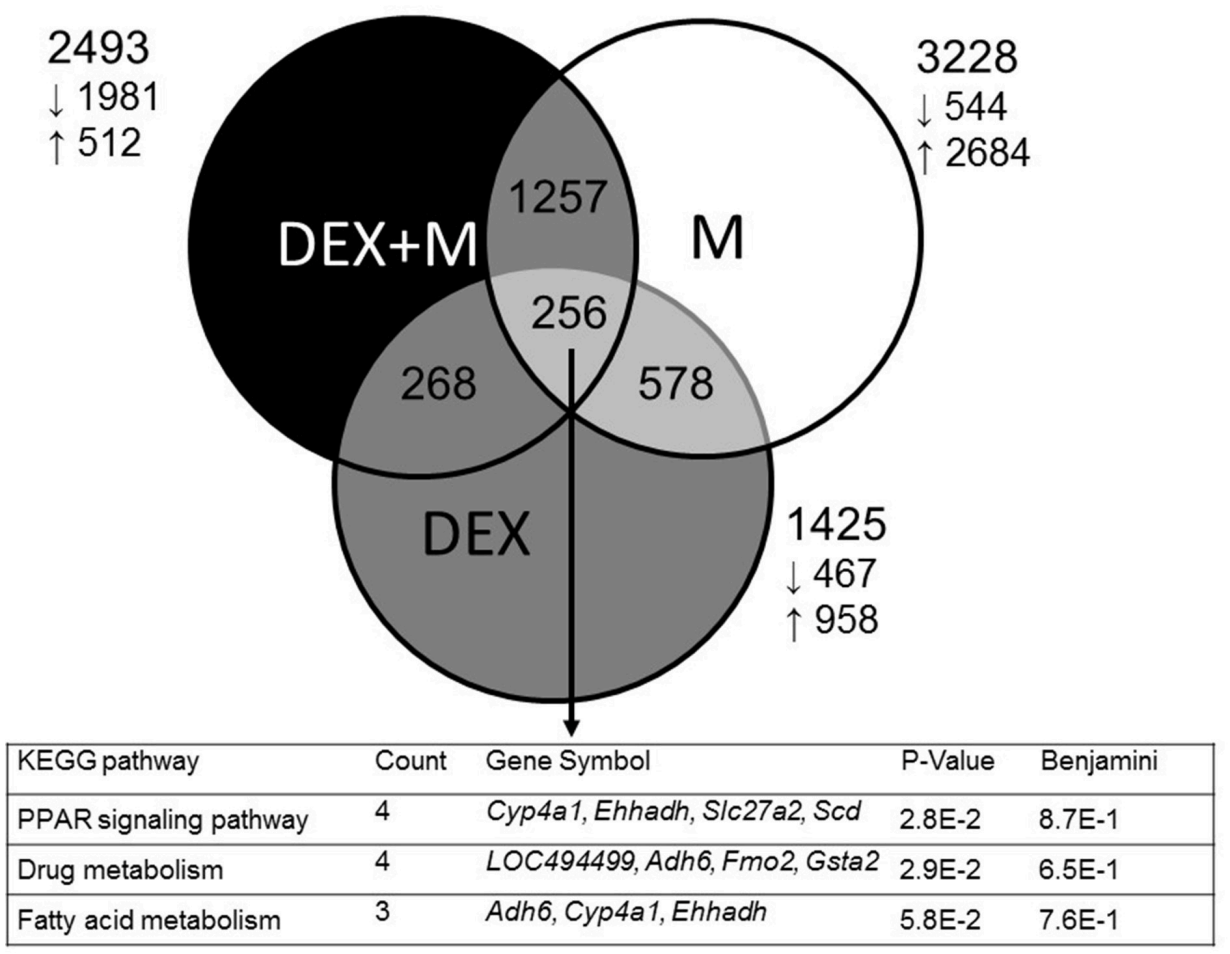
**Venn diagram depicting unique and shared (overlapping circles) sets of differential expressed genes (DEGs), between prenatal dexamethasone exposure (DEX, gray circle), melatonin treatment (M, white circle), and prenatal dexamethasone plus melatonin treatment (DEX+M, black circle)**. A total of three shared KEGG pathways are listed in the lower panel.

In the first series of experiments, we analyzed differential gene expression induced by DEX, melatonin, and DEX + melatonin in the 1-week-old male offspring kidney. Among the differential expressed genes (DEGs), a total of 1425 genes (958 up- and 467 down-regulated genes by DEX vs. control) met the selection criteria of (1) genes that changed by reads per kilobase of transcript per million mapped reads (RPKM) >0.3 and (2) minimum of two-fold difference in normalized read counts between group. Next, 3228 DEGs (2684 up- and 544 down-regulated genes) was observed in melatonin vs. control. In addition, there was a total of 2493 DEGs (512 up- and 1981 down-regulated genes) between DEX + melatonin and control group. Among them, 256 shared genes were identified (Table S2). Genes shared by different exposures were represented graphically by the Venn diagram shown in Figure 1.

We next used DAVID v6.7 (Dennis et al., 2003) to gain biological insight from our gene lists. We searched for KEGG pathways that are statistically enriched among three groups, and found 12, 55, and 36 such pathways in the DEX, melatonin, and DEX + melatonin group, respectively (Table S3). We observed that 5 KEGG pathways were shared by the three groups, including chemokine signaling pathway, axon guidance, Hedgehog signaling pathway, focal adhesion, and PPAR signaling pathway. Among 256 shared genes, we found three significantly regulated KEGG pathways, including PPAR signaling, drug metabolism, and fatty acid metabolism (Figure 1).

**Figure 2:**
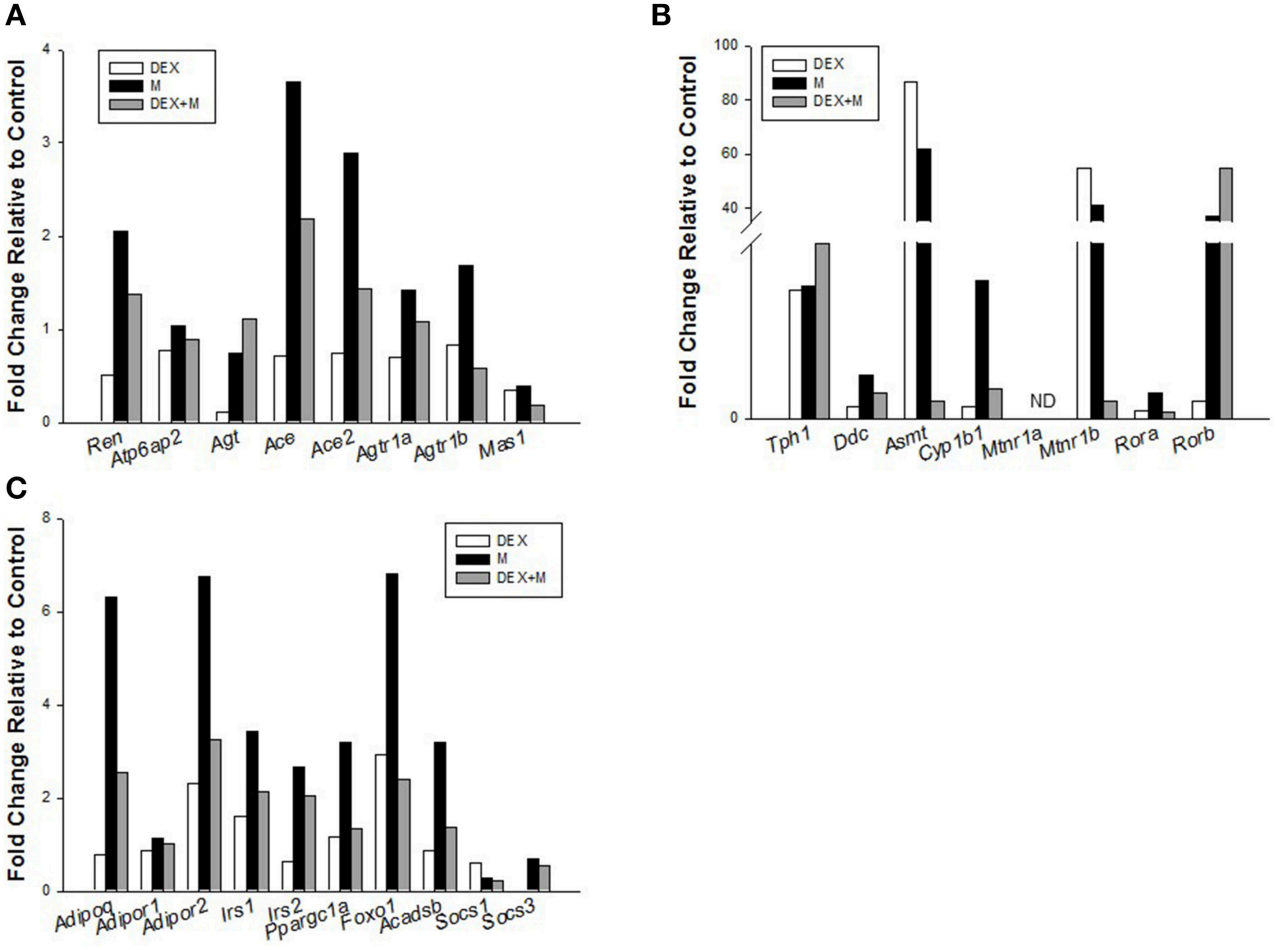
**Effects of prenatal dexamethasone exposure (DEX), melatonin treatment (M), and prenatal dexamethasone plus melatonin treatment (DEX+M) on gene expression in the (A) renin-angiotensin system, (B) synthesis, metabolism, and signaling pathway of melatonin, and (C) lipid metabolism and insulin signaling in the 1-week-old offspring kidney**.

We also examined three systems which might be involved in the DEX-induced programmed hypertension and protective effects of melatonin based on published data and our preliminary results. First, we looked at the renin-angiotensin system (RAS). RAS has been thought to contribute to programmed hypertension (Paixão and Alexander, 2013; Tain et al., 2014a). Our NGS data showed that RAS is a significantly regulated KEGG pathway in both melatonin and DEX + melatonin group (Table S2). The identified DEGs in RAS include *Agt* in DEX vs. control; *Ren, Ace, Ace2*, and *Mas1* in melatonin group vs. control; and *Ace* and *Mas1* in DEX + melatonin vs. control (Figure 2A). Next, we considered that maternal melatonin therapy might regulate the synthesis, metabolism, and signaling pathway of melatonin in the offspring kidney. We observed that genes involved in the biosynthetic pathway of melatonin were significantly upregulated, including *Tph1, Ddc*, and *Asmt* (Figure 2B). The *Cyp1b1*, a cytochrome P450 enzyme responsible for melatonin degradation, was also upregulated in melatonin group vs. control. Despite *Mtnr1a* (encoded for melatonin receptor 1) was undetectable, other melatonin receptors *Mtnr1b, Rora*, and *Rorb* were upregulated in melatonin group (Figure 2B). These data demonstrated that maternal melatonin treatment upregulates the synthesis, degradation, and downstream signaling pathway of melatonin in offspring kidney. Finally, mindful of the fact that the most common regulated KEGG pathways are related to lipid metabolism and insulin signaling, we also examined adiponectin (*Adipoq*) and its receptors (*Adipor1* and *Adipor2*), genes involved in fatty acid metabolism (*Acadsb*), and insulin signaling pathway (Figure 2C). Maternal melatonin therapy significantly increased the expression of *Adipoq* (Fold change [FC] = 6.3)*, Adipor2* (FC = 6.76)*, Irs1* (FC = 3.44)*, Irs2* (FC = 2.66)*, Ppargc1a* (FC = 3.2)*, Foxo1* (FC = 6.81), and *Acadsb* (FC = 3.2). Data by our NGS results described that maternal melatonin induced alterations of genes involved in insulin signaling and fatty acid degradation. Whether melatonin can regulate insulin sensitivity and fatty acid degradation to protect against programmed hypertension awaits further evaluation.

**Figure 3:**
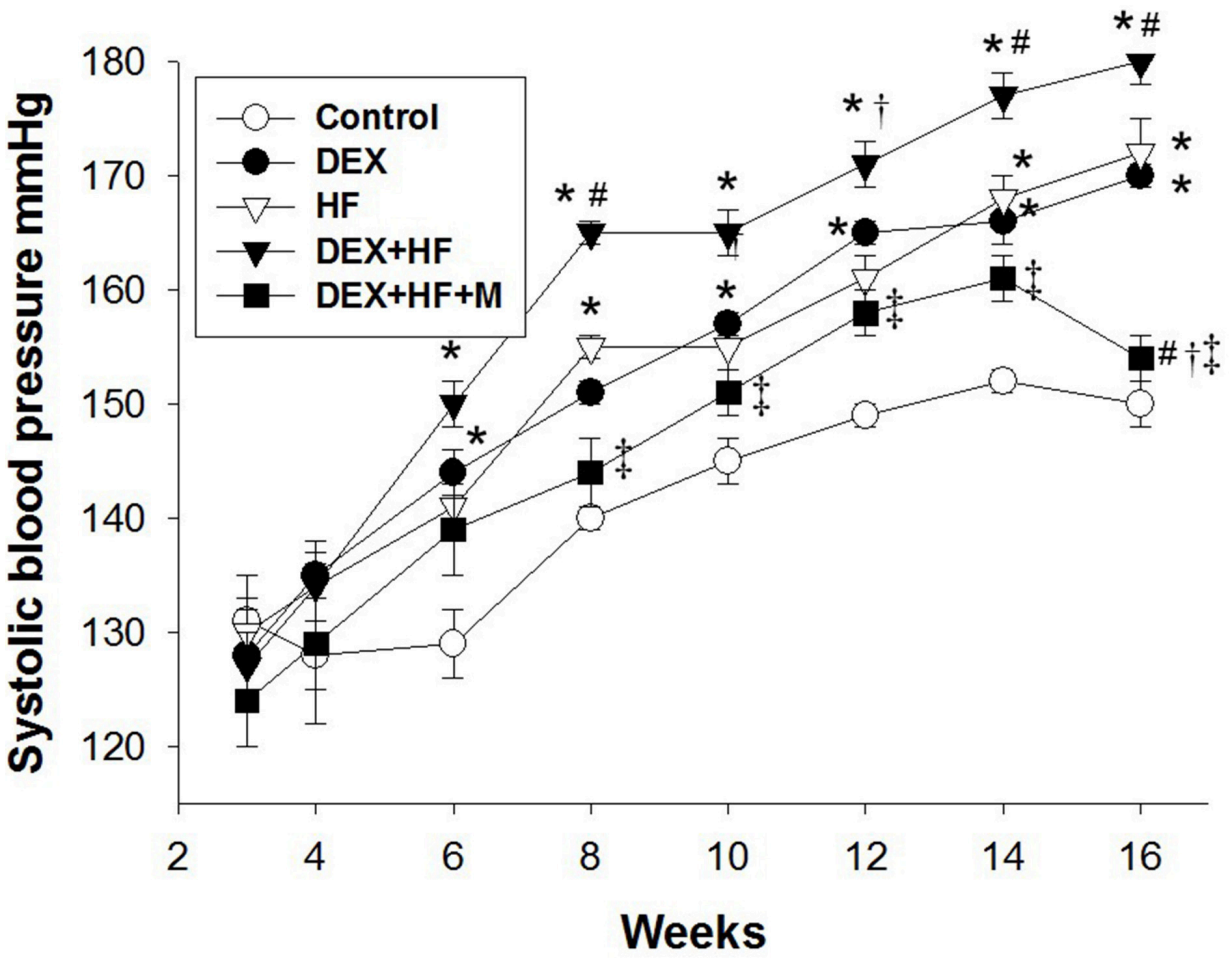
**Effects of prenatal dexamethasone exposure (DEX), postnatal high-fat diet (HF), prenatal DEX plus postnatal (DEX+HF), and maternal melatonin treatment (DEX+HF+M) on systolic blood pressure**. Blood pressure was measured in conscious rats by an indirect tail-cuff method between 3 and 16 weeks of age. *N* = 7–8/group. ^*^P < 0.05 vs. control; ^#^*P* < 0.05 vs. DEX; ^†^*P* < 0.05 vs. HF; ^‡^*P* < 0.05 vs. DEX+HF.

In the next series of experiments, we observed that male pup mortality rates, body weight, and kidney weight were not different among the groups (Table 1). The systolic and diastolic BPs and mean arterial pressure (MAP) of DEX group were significantly higher than those in the control group at 16 weeks of age (Figure 3). Postnatal HF intake caused a marked increase in BPs, and the systolic BP and MAP achieved were similar to those in DEX group. The DEX+HF group had the highest BPs compared to DEX, HF, and control group. These findings showed the synergistic interaction between the effects of prenatal DEX and postnatal HF on the elevation of BP. Of note, the increase of BPs was attenuated by maternal melatonin therapy (Figure 3).

**Table 1:** **Weights and functional parameters**.

	**Control *N* = 7**	**DEX *N* = 8**	**HF *N* = 7**	**DEX+HF *N* = 8**	**DEX+HF+M *N* = 8**
Mortality (%)	0	0	0	0	0
Body weight (BW) (g)	578±10	555±19	550±13	603±11	613±10
Left kidney weight (g)	1.93±0.06	2.05±0.06	1.98±0.05	2.1±0.07	2.14±0.08
Left kidney weight/100 g BW	0.33±0.01	0.37±0.01	0.36±0.01	0.35±0.011	0.35±0.01
Systolic blood pressure (mmHg)	150±2	170±58^*^	172±3^*^	180±2^*^^#^	154±2^#^^†^^‡^
Diastolic blood pressure (mmHg)	76±3	86±4	95±8^*^	109±4^*^^#^	76±2^†^^‡^
Mean arterial pressure (mmHg)	101±2	114±3^*^	121±2^*^	133±3^*^^#^	102±2^#^^†^^‡^

* *P < 0.05 vs. control*;

# *P < 0.05 vs. DEX*;

† *P < 0.05 vs. HF*;

‡ *P < 0.05 vs. DEX+HF*.

**Figure 4:**
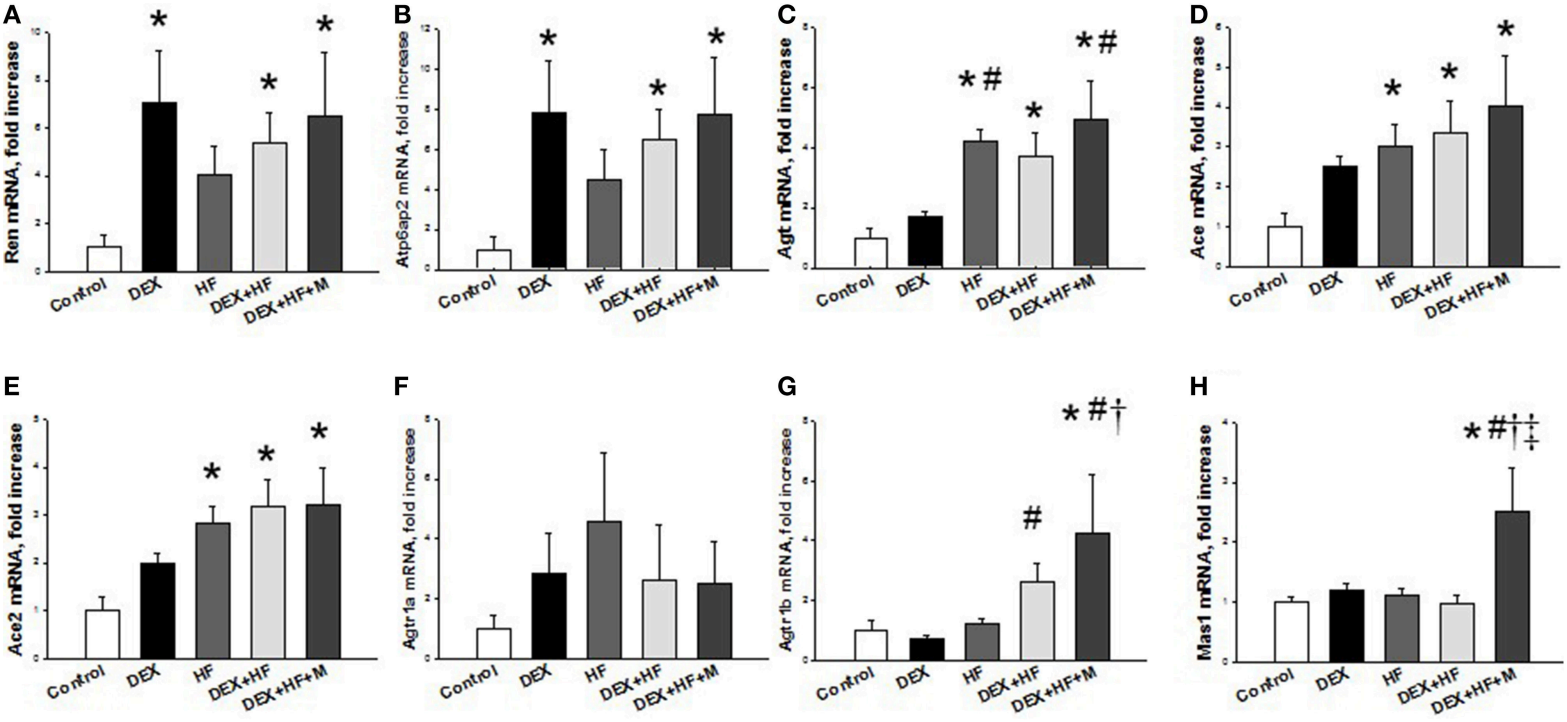
**Prenatal dexamethasone exposure (DEX), postnatal high-fat diet (HF), prenatal DEX plus postnatal (DEX+HF), and maternal melatonin treatment (DEX+HF+M) effects on gene expression of components of renin-angiotensin system, including (A) ***Ren***, (B) ***Atp6ap2***, (C) ***Agt***, (D) ***Ace***, (E) ***Ace2***, (F) ***Agtr1a***, (G) ***Agtr1b***, and (H) ***Mas1*** in the offspring kidney at 16 weeks of age. ***N*** = 6/group**. ^*^P < 0.05 vs. control; ^#^*P* < 0.05 vs. DEX; ^†^*P* < 0.05 vs. HF; ^‡^*P* < 0.05 vs. DEX+HF.

Next, we evaluated the mRNA expression of RAS components in the prenatal DEX plus postnatal HF two-hit model. As shown in Figure 4, prenatal DEX exposure significantly upregulated mRNA expression of *Ren* (FC = 7.05) and *Atp6ap2* (FC = 7.84) in the 16-week-old kidneys. Postnatal HF diets induced *Agt* (FC = 4.22)*, Ace* (FC = 3), and *Ace2* (FC = 2.84) mRNA expression in kidney. DEX+HF led to increased *Ren* (FC = 5.35), *Atp6ap2* (FC = 6.45)*, Agt* (FC = 3.73)*, Ace* (FC = 3.35), and *Ace2* (FC = 3.19) in the kidney. Maternal melatonin therapy increased renal *Ren* (FC = 6.46), *Atp6ap2* (FC = 7.71), *Agt* (FC = 4.96)*, Ace* (FC = 4.01), *Ace2* (FC = 3.21), *Agtr1b* (FC = 4.25), and *Mas1* (FC = 2.51) mRNA expression in the DEX+HF+M group.

**Figure 5:**
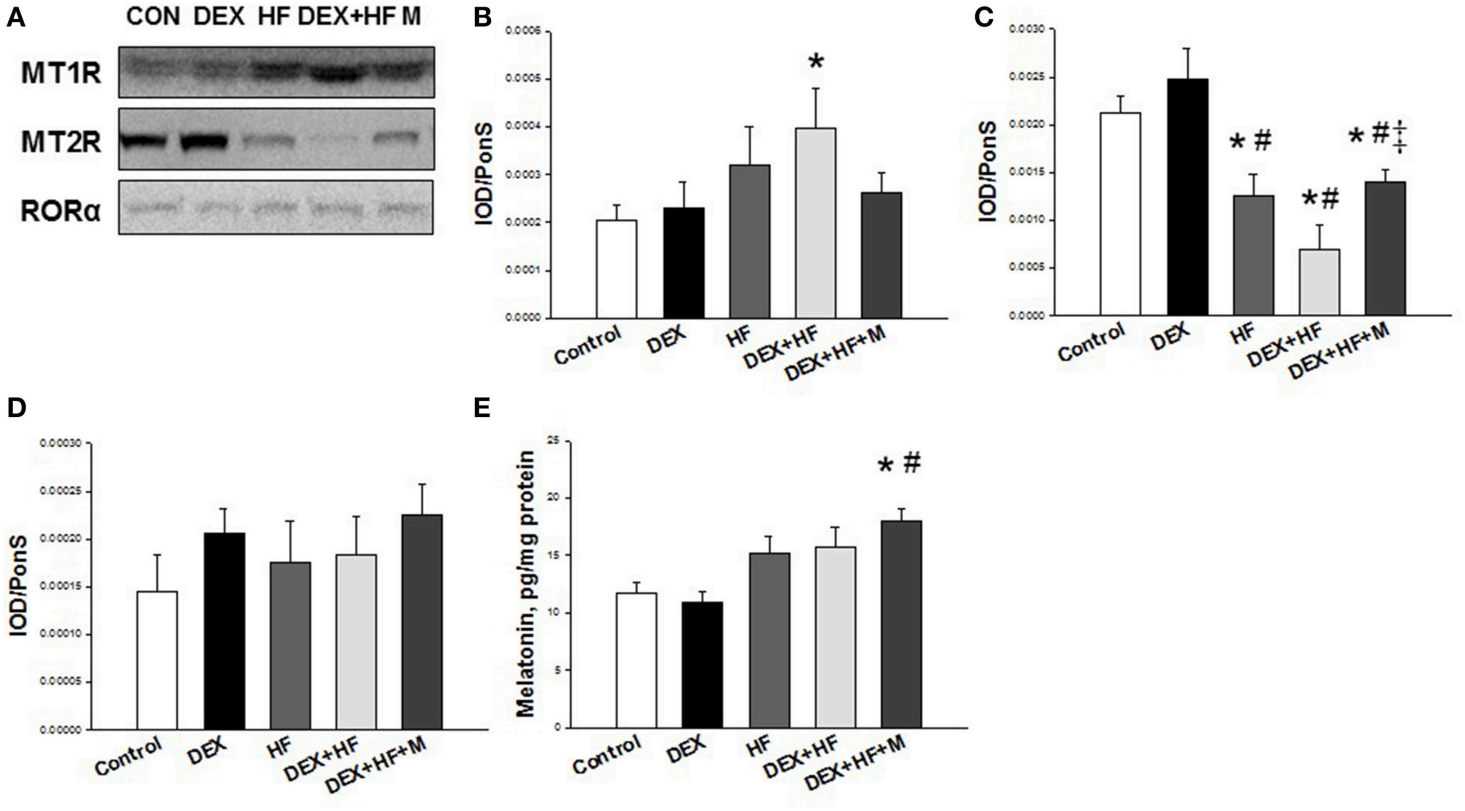
**Representative western blots showing (A) melatonin receptor-1 (MT1) (37 kDa), and −2 (MT2) (40 kDa), and RORα (59 kDa) bands in control, prenatal dexamethasone exposure (DEX), postnatal high-fat diet (HF), prenatal DEX plus postnatal (DEX+HF), and melatonin treatment (DEX+HF+M) offspring rats at 16 weeks of age**. Relative abundance of renal cortical **(B)** MT1, **(C)** MT2, and **(D)** RORα as quantified. **(E)** Effect of DEX, HF, and melatonin on renal melatonin level. *N* = 6/group. ^*^P < 0.05 vs. control; ^#^*P* < 0.05 vs. DEX; ^‡^*P* < 0.05 vs. DEX+HF.

Furthermore, we examined whether melatonin and its receptors were altered in the programmed hypertension in response to DEX, HF, and melatonin. As shown in Figure 5, renal MT1 level was high in the DEX+HF group (+95.3%) vs. control. MT2 protein abundance in the kidney was lower in the HF (−40.4%), DEX+HF (−67.4%), and DEX+HF+M groups (−34.2%) than that in control and DEX group. RORα protein level was not different among the five groups (Figure 5). However, maternal melatonin therapy increased renal melatonin level compared to control and the DEX group (Figure 5E).

## Discussion

This study provides insight into a novel mechanism by which maternal melatonin therapy prevents prenatal DEX and postnatal HF intake synergistically induced programmed hypertension in adult male offspring. The main findings can be summarized as follow: (1) a total of 256 differential expressed genes (DEGs) were shared by prenatal DEX, melatonin, and DEX + melatonin exposure in the 1-week-old male offspring kidney; (2) maternal melatonin treatment upregulates the synthesis, degradation, and downstream signaling pathway of melatonin in the offspring kidney; (3) prenatal DEX and postnatal HF diet synergistically induced programmed hypertension in adult offspring, which maternal melatonin prevented; and (4) the protective effects of melatonin on programmed hypertension in this two-hit model is associated with alterations of genes in RAS and melatonin receptors.

Despite the well-documented anti-hypertensive effects of melatonin in adults (Simko and Paulis, 2007; Reiter et al., 2010), limited data are available regarding dams under melatonin therapy during pregnancy and lactation causing long-term BP-lowering effects in their adult offspring, especially focused on renal programming. To the best of our knowledge, our study is the first to show that maternal melatonin therapy can prevent programmed hypertension in adult rats synergistically induced by prenatal DEX and postnatal HF diet. It is consistent with our recent reports showing that maternal melatonin therapy prevents programmed hypertension in a variety of programming models (Tain et al., 2014a,c,d; Wu et al., 2014).

Given that nephrogenesis occurs from late gestation to postnatal week 1–2 in rats, maternal melatonin treatment used in this study covered the whole nephrogenesis period. In agreement with previous studies (Korkmaz et al., 2012; Tain et al., 2014b), we observed that 6 weeks of maternal melatonin therapy is likely to serve as an inducer of gene expression in the developing kidney. Regardless of whether melatonin therapy has a benign safety profile for pregnant women (Chen et al., 2013), whether maternal melatonin therapy might cause long-term epigenetic changes leading to adverse effects in adult offspring remains to be clarified. Our data also indicate that early melatonin therapy may deprogram high-fat induced programmed process and prevent the development of hypertension during later life.

Melatonin has been proposed to prevent obesity-related disorders (Cipolla-Neto et al., 2014). Our NGS data demonstrated that maternal melatonin can upregulate several genes involved in energy control in offspring kidney, which may increase insulin sensitivity and fatty acid degradation. It is possible that early melatonin therapy elicit programmed process for the regulation of fatty acids in the offspring that helps to moderate the effects of postnatally HF diet.

Next, some particular candidate genes/pathways related to the programmed hypertension have been studied in different models of developmental programming, such as the RAS (Ritz et al., 2011; Paixão and Alexander, 2013). Indeed, the RAS plays an essential role in kidney development as well as in programmed hypertension (Manning, 2004; Yosypiv, 2014). Our results showed that maternal melatonin therapy increased *Agtr1b* (encoded for AT2R) and *Mas1* (encoded for Mas) mRNA expression in the kidney. It is well-established that the signaling triggered by AT2R or Mas activation represents an endogenous counter-regulatory pathway within the RAS, that is in opposition to the vasoconstrictor arm of the RAS (Chappell et al., 2014). Thus, one might expect maternal melatonin therapy to adjust the RAS in a way that opposes the development of hypertension in the DEX+HF+M group, similar to our previous report in a rat model of maternal caloric restriction-induced programmed hypertension (Hsu et al., 2014).

Another possible protective mechanism of melatonin on programmed hypertension is related to its regulation on the melatonin pathway in the offspring kidney. Our NGS data demonstrated that maternal melatonin therapy programmed synthesis, metabolism, and signaling pathway of melatonin in the developing offspring kidney. Importantly, maternal melatonin increased renal melatonin level, and restored two-hit insults induced increased MT1 and decreased MT2 protein levels in adult offspring. MT1 has been found to mediate vascular constriction, while MT2 mediates vasodilatation (Doolen et al., 1998). These findings indicate early melatonin therapy elicits a persistent effect on the melatonin level and its receptors, at least in part, to prevent prenatal DEX and postnatal HF synergistically induced programmed hypertension. Given that MT2 defects link to the development of diabetes (Bonnefond et al., 2012), and that a strong association between HF intake and diabetes, whether early targeting on melatonin/MT2 pathway can reduce offspring vulnerability to postnatal HF-induced programming deserves further study. Furthermore, prenatal DEX and postnatal HF induced programmed hypertension in adult offspring, while both altered gene expression of the RAS components and melatonin receptors differentially. These observations suggest that different insults might generate a complex set of programming responses; some pathways have commonality, but others may not, despite the manifests of same phenotype-hypertension. Thus, the implications of other mechanisms involved in melatonin-induced deprogramming deserve further clarification.

In NGS dataset, we observed two important KEGG pathways shared by DEX, M, and DEX+M group, including PPAR signaling and fatty acid metabolism. We have recently shown that PPAR signaling pathway is identified as a common pathway related to programmed hypertension in the caloric restriction, maternal diabetes, and high-fructose intake models (Tain et al., 2015). The PPAR signaling pathway has been well-studied in established hypertension and is becoming a therapeutic target for hypertension (Duan et al., 2009). Next, fatty acid metabolism was identified as a commonly regulated KEGG pathway in both the caloric restriction and high-fructose intake model in our previous study (Tain et al., 2015). Here we demonstrated that maternal melatonin induced increases in fatty acid degradation and insulin sensitivity. Therefore, additional studies are required to unravel whether protective effects of melatonin on programmed hypertension are via the PPAR signaling and fatty acid metabolism pathways in other programmed models.

In conclusion, postnatal HF diet enhanced prenatal DEX-induced programmed hypertension in adult offspring, which can be prevented by maternal melatonin therapy. Maternal melatonin therapy has long-term effects on renal programming, including regulation of the synthesis and signaling pathway of melatonin and increases of *Agtr1b* and *Mas1* mRNA expression. By providing candidate genes and pathways responsible for programmed hypertension, our results are of significance to the development of novel interventions in the prevention of renal programming and hypertension in children exposed to prenatal glucocorticoid and postnatal high-fat intake.

## Author contributions

Conception and design: YT, JS, CH, LH. Animal treatment, collection, and measurements: YT, HY, CC, MT, YL, LH. Analysis and interpretation of data: YT, JS, CH, LH. Drafting and/or revising the article critically for important intellectual content: YT, JS, HY, CC, MT, CH, YL, LH. Approved the final version of the manuscript: YT, JS, HY, CC, MT, CH, YL, LH.

## Funding

This work was supported by Grant CMRPG8C0042 from Chang Gung Memorial Hospital, Kaohsiung, Taiwan, and the Grant MOST 104-2314-B-182-056-MY3 from the Ministry of Science and Technology, Taiwan.

### Conflict of interest statement

The authors declare that the research was conducted in the absence of any commercial or financial relationships that could be construed as a potential conflict of interest.
