# Supplementary material for: Maternal Melatonin Therapy Rescues Prenatal Dexamethasone and Postnatal High-Fat Diet Induced Programmed Hypertension in Male Rat Offspring
Source: Front Physiol. 2015 Dec 11;6:377. doi: 10.3389/fphys.2015.00377 (PMC4675845; doi:10.3389/fphys.2015.00377)
Supplement: Supplementary file 1 [file Table1.DOCX]

Supplementary Material

Maternal Melatonin Therapy Rescues Prenatal Dexamethasone and Postnatal High-Fat Diet Induced Programmed Hypertension in Male Rat Offspring

You-Lin Tain1,2, Jiunn-Ming Sheen1, Hong-Ren Yu1, Chih-Cheng Chen1, Mao-Meng Tiao1, Chien-Ning Hsu,3,4 Yu-Ju Lin5, Kuang-Che Kuo, Li-Tung Huang1,6*

*** Correspondence:** Li-Tung Huang: litung.huang@gmail.com

# Supplementary Table 1

**Table S1 qPCR primers sequences**

| Gene | Forward | Reverse |
| --- | --- | --- |
| *Ren* | 5 aacattaccagggcaactttcact 3 | 5 acccccttcatggtgatctg 3 |
| *Atp6ap2* | 5 gaggcagtgaccctcaacat 3 | 5 ccctcctcacacaacaaggt 3 |
| *Agt* | 5 gcccaggtcgcgatgat 3 | 5 tgtacaagatgctgagtgaggcaa 3 |
| *Ace* | 5 caccggcaaggtctgctt 3 | 5 cttggcatagtttcgtgaggaa 3 |
| *Ace2* | 5 acccttcttacatcagccctactg 3 | 5 tgtccaaaacctaccccacatat 3 |
| *Agtr1a* | 5 gctgggcaacgagtttgtct 3 | 5 cagtccttcagctggatcttca 3 |
| *Agtr1b* | 5 caatctggctgtggctgactt 3 | 5 tgcacatcacaggtccaaaga 3 |
| *Mas1* | 5 catctctcctctcggctttgtg 3 | 5 cctcatccggaagcaaagg 3 |
| *Rn18s* | 5 gccgcggtaattccagctcca 3 | 5 cccgcccgctcccaagatc 3 |

*Ren*= Renin, *Atp6ap2*= Prorenin receptor (PRR), *Agt*= Angiotensinogen (AGT), *Ace*= Angiotensin converting enzyme (ACE), *Agtr1a* = Angiotensin II type 1 receptor (AT1R), *Agtr1b* = Angiotensin II type 2 receptor (AT2R), *Mtnr1a*= melatonin receptor 1 (MT1), *Mtnr1b*= melatonin receptor 2 (MT2), *Rora*= RORα, *Rn18s* = 18S ribosomal RNA (r18S).

# Supplementary Table 2

**Table S2. Fold changes in shared genes in the kidney of offspring at 1 week of age exposed to dexamethasone (DEX), melatonin, and DEX+melatonin**

| Gene ID | Gene Symbol | Melatonin | Dex | Dex+Melatonin |
| --- | --- | --- | --- | --- |
| ENSRNOG00000000201 | *Gsta2* | 2.02 | 0.30 | 0.15 |
| ENSRNOG00000000407 | *Dcbld1* | 4.83 | 2.28 | 0.47 |
| ENSRNOG00000000499 | *Tcp11* | 0.30 | 2.15 | 7.12 |
| ENSRNOG00000000547 | *Tspyl4* | 8.20 | 3.13 | 0.38 |
| ENSRNOG00000001092 | *Kl* | 5.87 | 0.42 | 0.07 |
| ENSRNOG00000001197 | *Pdxk* | 2.05 | 0.47 | 0.23 |
| ENSRNOG00000001270 | *RGD1310788* | 8.66 | 4.17 | 0.48 |
| ENSRNOG00000001335 | *Zkscan1* | 6.42 | 2.52 | 0.39 |
| ENSRNOG00000001338 | *Hpd* | 2.76 | 0.29 | 0.11 |
| ENSRNOG00000001518 | *Itga6* | 5.52 | 2.03 | 0.37 |
| ENSRNOG00000001724 | *LOC678704* | 10.69 | 2.40 | 0.22 |
| ENSRNOG00000001747 | *Pak2* | 4.57 | 2.26 | 0.50 |
| ENSRNOG00000001766 | *Tfrc* | 5.61 | 2.07 | 0.37 |
| ENSRNOG00000001770 | *Ehhadh* | 4.11 | 0.49 | 0.12 |
| ENSRNOG00000002053 | *F1M3H3_RAT* | 5.49 | 2.05 | 0.37 |
| ENSRNOG00000002198 | *LOC685352* | 8.71 | 4.30 | 0.49 |
| ENSRNOG00000002276 | *LOC100359714* | 7.64 | 3.17 | 0.41 |
| ENSRNOG00000002321 | *RGD1563242* | 10.65 | 3.72 | 0.35 |
| ENSRNOG00000002332 | *MSPD1_RAT* | 6.03 | 2.18 | 0.36 |
| ENSRNOG00000002461 | *Nid1* | 6.06 | 2.18 | 0.36 |
| ENSRNOG00000002463 | *LOC682752* | 10.63 | 3.49 | 0.33 |
| ENSRNOG00000002509 | *Gnl3l* | 6.13 | 2.48 | 0.40 |
| ENSRNOG00000002519 | *Magt1* | 9.25 | 2.35 | 0.25 |
| ENSRNOG00000002969 | *Itpkb* | 9.89 | 2.64 | 0.27 |
| ENSRNOG00000003038 | *Sft2d2* | 17.10 | 2.13 | 0.12 |
| ENSRNOG00000003472 | *Atp11c-ps1* | 5.47 | 2.13 | 0.39 |
| ENSRNOG00000003510 | *Fmo2* | 3.23 | 0.47 | 0.15 |
| ENSRNOG00000003759 | *Galc* | 6.35 | 2.34 | 0.37 |
| ENSRNOG00000003865 | *Tmigd1* | 4.48 | 0.31 | 0.07 |
| ENSRNOG00000004009 | *Xpnpep2* | 3.12 | 0.43 | 0.14 |
| ENSRNOG00000004222 | *S100g* | 2.70 | 0.37 | 0.14 |
| ENSRNOG00000004302 | *Pah* | 3.27 | 0.45 | 0.14 |
| ENSRNOG00000004448 | *RGD1307051* | 6.82 | 2.00 | 0.29 |
| ENSRNOG00000004531 | *Tceanc* | 4.87 | 2.30 | 0.47 |
| ENSRNOG00000004563 | *Sec24a* | 4.92 | 2.38 | 0.48 |
| ENSRNOG00000004888 | *Spred2* | 6.69 | 2.66 | 0.40 |
| ENSRNOG00000004958 | *RGD1304605* | 5.56 | 0.36 | 0.06 |
| ENSRNOG00000004959 | *Actr2* | 4.81 | 2.00 | 0.42 |
| ENSRNOG00000005070 | *Spopl* | 6.92 | 2.08 | 0.30 |
| ENSRNOG00000005131 | *Lin7c* | 5.09 | 2.13 | 0.42 |
| ENSRNOG00000005417 | *Zhx2* | 4.95 | 2.32 | 0.47 |
| ENSRNOG00000005447 | *RGD1311564* | 10.12 | 2.07 | 0.20 |
| ENSRNOG00000005544 | *D3ZJ09_RAT* | 4.05 | 2.02 | 0.50 |
| ENSRNOG00000005933 | *Yap1* | 5.32 | 2.29 | 0.43 |
| ENSRNOG00000006338 | *Lrp6* | 6.35 | 2.08 | 0.33 |
| ENSRNOG00000006436 | *Spty2d1* | 4.79 | 2.35 | 0.49 |
| ENSRNOG00000006628 | *Dusp16* | 5.26 | 2.59 | 0.49 |
| ENSRNOG00000006753 | *Tgm3* | 2.52 | 0.45 | 0.18 |
| ENSRNOG00000006787 | *Dhcr24* | 9.33 | 2.11 | 0.23 |
| ENSRNOG00000007445 | *D3ZK62_RAT* | 4.79 | 2.12 | 0.44 |
| ENSRNOG00000007632 | *Zmynd17* | 0.21 | 0.44 | 2.10 |
| ENSRNOG00000007804 | *C1galt1* | 6.76 | 2.32 | 0.34 |
| ENSRNOG00000007985 | *D4ABH6_RAT* | 4.08 | 2.02 | 0.49 |
| ENSRNOG00000007990 | *Adipor2* | 6.76 | 2.32 | 0.34 |
| ENSRNOG00000008271 | *Fam91a1* | 6.22 | 2.04 | 0.33 |
| ENSRNOG00000008895 | *Hnf4a* | 7.99 | 2.15 | 0.27 |
| ENSRNOG00000009019 | *Slc6a6* | 10.09 | 2.05 | 0.20 |
| ENSRNOG00000009506 | *Mre11a* | 6.00 | 2.62 | 0.44 |
| ENSRNOG00000009565 | *Pdk4* | 5.76 | 2.65 | 0.46 |
| ENSRNOG00000009597 | *Cyp4a1* | 5.74 | 0.46 | 0.08 |
| ENSRNOG00000009613 | *D4ADA8_RAT* | 6.58 | 2.07 | 0.31 |
| ENSRNOG00000009944 | *LOC314407* | 10.86 | 3.05 | 0.28 |
| ENSRNOG00000010128 | *S27A2_RAT* | 2.83 | 0.41 | 0.14 |
| ENSRNOG00000010388 | *Slc21a4* | 3.36 | 0.50 | 0.15 |
| ENSRNOG00000010813 | *Tspan14* | 5.05 | 2.10 | 0.42 |
| ENSRNOG00000011009 | *Cmtm4* | 4.69 | 2.09 | 0.45 |
| ENSRNOG00000011022 | *Mep1a* | 2.91 | 0.40 | 0.14 |
| ENSRNOG00000011040 | *-* | 4.40 | 2.05 | 0.47 |
| ENSRNOG00000011136 | *Osr2* | 0.42 | 5.15 | 12.25 |
| ENSRNOG00000011560 | *Mtmr9* | 5.74 | 2.36 | 0.41 |
| ENSRNOG00000011630 | *Ak3l1* | 10.30 | 3.11 | 0.30 |
| ENSRNOG00000011692 | *Dkk1* | 2.17 | 4.89 | 2.25 |
| ENSRNOG00000011696 | *Lifr* | 2.81 | 0.45 | 0.16 |
| ENSRNOG00000011833 | *-* | 0.13 | 0.42 | 3.15 |
| ENSRNOG00000011859 | *Eif5a2* | 8.19 | 3.66 | 0.45 |
| ENSRNOG00000011861 | *Aadat* | 2.27 | 0.39 | 0.17 |
| ENSRNOG00000011927 | *SDC3_RAT* | 8.46 | 2.93 | 0.35 |
| ENSRNOG00000012337 | *Pde1c* | 2.87 | 0.38 | 0.13 |
| ENSRNOG00000012436 | *Adh6* | 3.90 | 0.48 | 0.12 |
| ENSRNOG00000013082 | *LCAP_RAT* | 5.75 | 2.50 | 0.43 |
| ENSRNOG00000013213 | *Epha4* | 4.47 | 2.05 | 0.46 |
| ENSRNOG00000013240 | *Ptger4* | 2.47 | 0.46 | 0.19 |
| ENSRNOG00000013279 | *Scd* | 10.78 | 3.19 | 0.30 |
| ENSRNOG00000013310 | *-* | 0.17 | 0.38 | 2.21 |
| ENSRNOG00000013443 | *Tm9sf3* | 6.79 | 2.71 | 0.40 |
| ENSRNOG00000013469 | *LOC100362805* | 5.71 | 2.30 | 0.40 |
| ENSRNOG00000013661 | *Kif26a* | 2.10 | 0.45 | 0.21 |
| ENSRNOG00000014028 | *F1LXJ6_RAT* | 3.63 | 0.43 | 0.12 |
| ENSRNOG00000014166 | *Smoc2* | 3.08 | 0.32 | 0.10 |
| ENSRNOG00000014234 | *Hif1an* | 5.19 | 2.16 | 0.42 |
| ENSRNOG00000014511 | *Alg10* | 7.55 | 3.46 | 0.46 |
| ENSRNOG00000014685 | *Nlrp6* | 3.73 | 0.44 | 0.12 |
| ENSRNOG00000014866 | *Pign* | 7.08 | 2.04 | 0.29 |
| ENSRNOG00000014948 | *Osgin1* | 2.23 | 0.45 | 0.20 |
| ENSRNOG00000014976 | *Acsm2* | 3.85 | 0.45 | 0.12 |
| ENSRNOG00000015038 | *Adam10* | 9.28 | 2.29 | 0.25 |
| ENSRNOG00000015080 | *Wdfy1* | 5.77 | 2.01 | 0.35 |
| ENSRNOG00000015177 | *Unc84b* | 5.20 | 2.20 | 0.42 |
| ENSRNOG00000015557 | *Umod* | 2.02 | 0.35 | 0.17 |
| ENSRNOG00000015575 | *LOC312502* | 5.06 | 2.33 | 0.46 |
| ENSRNOG00000015605 | *Ptprk* | 7.36 | 2.52 | 0.34 |
| ENSRNOG00000016412 | *Fxyd6* | 4.56 | 2.08 | 0.46 |
| ENSRNOG00000016671 | *Dtna* | 2.38 | 5.10 | 2.14 |
| ENSRNOG00000016715 | *Kif11* | 4.55 | 2.25 | 0.50 |
| ENSRNOG00000017078 | *Sepn1* | 11.11 | 3.31 | 0.30 |
| ENSRNOG00000017172 | *Fam125b* | 8.71 | 2.42 | 0.28 |
| ENSRNOG00000017434 | *Mgat3* | 12.36 | 2.70 | 0.22 |
| ENSRNOG00000017625 | *Htr2b* | 0.37 | 0.17 | 0.46 |
| ENSRNOG00000017671 | *Rasa3* | 4.94 | 2.22 | 0.45 |
| ENSRNOG00000017788 | *Katnal2* | 0.33 | 2.83 | 8.64 |
| ENSRNOG00000017964 | *Slc22a25* | 3.57 | 0.46 | 0.13 |
| ENSRNOG00000018109 | *Clic4* | 7.25 | 3.36 | 0.46 |
| ENSRNOG00000018123 | *Ccny* | 4.40 | 2.02 | 0.46 |
| ENSRNOG00000018220 | *Pde4dip* | 5.38 | 2.08 | 0.39 |
| ENSRNOG00000018338 | *Vwa1* | 9.02 | 3.10 | 0.34 |
| ENSRNOG00000018402 | *Apoc2* | 3.85 | 0.50 | 0.13 |
| ENSRNOG00000018420 | *Slc22a7* | 2.75 | 0.43 | 0.16 |
| ENSRNOG00000018588 | *Sox4* | 6.02 | 2.17 | 0.36 |
| ENSRNOG00000018714 | *Arl5b* | 7.77 | 3.61 | 0.46 |
| ENSRNOG00000019372 | *Pc* | 3.02 | 0.45 | 0.15 |
| ENSRNOG00000019470 | *Hao2* | 2.85 | 0.49 | 0.17 |
| ENSRNOG00000019799 | *Pcdhgc3* | 7.23 | 2.04 | 0.28 |
| ENSRNOG00000020011 | *Q66HF5_RAT* | 5.68 | 2.21 | 0.39 |
| ENSRNOG00000020253 | *RAB1B_RAT* | 5.03 | 2.34 | 0.46 |
| ENSRNOG00000020261 | *Fam53c* | 11.86 | 2.78 | 0.23 |
| ENSRNOG00000020284 | *Prkar2a* | 5.08 | 2.03 | 0.40 |
| ENSRNOG00000020532 | *Kcnq1* | 8.90 | 2.48 | 0.28 |
| ENSRNOG00000020538 | *D3Z8F4_RAT* | 7.17 | 2.10 | 0.29 |
| ENSRNOG00000021203 | *Atl3* | 9.49 | 2.29 | 0.24 |
| ENSRNOG00000021214 | *Slc22a9* | 2.30 | 0.31 | 0.13 |
| ENSRNOG00000021265 | *Cds2* | 5.61 | 2.29 | 0.41 |
| ENSRNOG00000021292 | *-* | 11.45 | 3.15 | 0.28 |
| ENSRNOG00000022101 | *Crabp2* | 0.36 | 2.08 | 5.76 |
| ENSRNOG00000022163 | *D3ZUE9_RAT* | 0.48 | 2.05 | 4.26 |
| ENSRNOG00000022227 | *-* | 2.36 | 5.89 | 2.50 |
| ENSRNOG00000022309 | *D3ZRU8_RAT* | 5.31 | 2.11 | 0.40 |
| ENSRNOG00000022561 | *D3ZV58_RAT* | 4.97 | 2.16 | 0.43 |
| ENSRNOG00000022710 | *Prrg4* | 5.74 | 2.13 | 0.37 |
| ENSRNOG00000022802 | *Tmem184b* | 8.98 | 3.24 | 0.36 |
| ENSRNOG00000023725 | *LOC689756* | 8.21 | 2.34 | 0.29 |
| ENSRNOG00000024089 | *Fndc3b* | 7.22 | 2.44 | 0.34 |
| ENSRNOG00000024533 | *Aer61* | 4.89 | 2.16 | 0.44 |
| ENSRNOG00000024757 | *RGD1310444* | 9.12 | 2.05 | 0.23 |
| ENSRNOG00000024848 | *FIBA_RAT* | 2.89 | 0.42 | 0.15 |
| ENSRNOG00000025042 | *Pde3a* | 6.05 | 2.14 | 0.35 |
| ENSRNOG00000025372 | *Glce* | 9.54 | 2.15 | 0.23 |
| ENSRNOG00000025863 | *LOC100362395* | 0.26 | 0.09 | 0.36 |
| ENSRNOG00000026120 | *Fam8a1* | 6.28 | 2.19 | 0.35 |
| ENSRNOG00000026965 | *Tmem140* | 2.52 | 0.50 | 0.20 |
| ENSRNOG00000027164 | *LOC100302372* | 8.55 | 2.11 | 0.25 |
| ENSRNOG00000027320 | *Eif2c1* | 6.08 | 2.61 | 0.43 |
| ENSRNOG00000027507 | *LOC100361333* | 6.20 | 2.77 | 0.45 |
| ENSRNOG00000028129 | *Fktn* | 8.21 | 3.73 | 0.45 |
| ENSRNOG00000028387 | *E9PTK5_RAT* | 8.17 | 2.59 | 0.32 |
| ENSRNOG00000028730 | *D3ZI71_RAT* | 0.15 | 0.41 | 2.80 |
| ENSRNOG00000028899 | *Zbtb33* | 5.63 | 2.50 | 0.44 |
| ENSRNOG00000029115 | *-* | 0.28 | 0.13 | 0.47 |
| ENSRNOG00000029216 | *Dgcr2* | 8.33 | 2.13 | 0.26 |
| ENSRNOG00000029409 | *Gstm6l* | 8.06 | 2.51 | 0.31 |
| ENSRNOG00000029811 | *Kcne2* | 0.45 | 0.21 | 0.46 |
| ENSRNOG00000029861 | *LOC494499* | 2.89 | 0.31 | 0.11 |
| ENSRNOG00000030731 | *LOC100359529* | 3.24 | 0.34 | 0.11 |
| ENSRNOG00000031069 | *D4A9A7_RAT* | 4.75 | 2.28 | 0.48 |
| ENSRNOG00000031353 | *Ust5r* | 2.80 | 0.36 | 0.13 |
| ENSRNOG00000031658 | *F1LVV8_RAT* | 5.14 | 2.49 | 0.49 |
| ENSRNOG00000031833 | *Sult1c2* | 4.18 | 0.38 | 0.09 |
| ENSRNOG00000031890 | *Ncam1* | 4.41 | 2.06 | 0.47 |
| ENSRNOG00000032006 | *F1M5G3_RAT* | 4.16 | 2.08 | 0.50 |
| ENSRNOG00000032284 | *D3ZNN2_RAT* | 0.48 | 0.13 | 0.27 |
| ENSRNOG00000032720 | *Rpl10l* | 5.54 | 2.75 | 0.50 |
| ENSRNOG00000032723 | *Eftud1* | 5.51 | 2.40 | 0.44 |
| ENSRNOG00000032768 | *D3Z9G8_RAT* | 6.50 | 2.60 | 0.40 |
| ENSRNOG00000032834 | *Hspa13* | 8.54 | 2.82 | 0.33 |
| ENSRNOG00000033517 | *LOC100360791* | 0.13 | 0.48 | 3.74 |
| ENSRNOG00000033596 | *F1M1L3_RAT* | 0.23 | 0.48 | 2.11 |
| ENSRNOG00000033660 | *Slc22a13* | 2.26 | 0.31 | 0.14 |
| ENSRNOG00000034324 | *U1* | 0.17 | 0.38 | 2.18 |
| ENSRNOG00000034397 | *U6* | 0.26 | 5.84 | 22.72 |
| ENSRNOG00000034511 | *U6* | 0.09 | 0.40 | 4.45 |
| ENSRNOG00000034552 | *U1* | 0.17 | 0.38 | 2.18 |
| ENSRNOG00000034727 | *SNORA32* | 0.06 | 5.13 | 89.81 |
| ENSRNOG00000034806 | *U1* | 0.17 | 0.38 | 2.18 |
| ENSRNOG00000034861 | *U1* | 0.17 | 0.38 | 2.22 |
| ENSRNOG00000034975 | *U6* | 0.26 | 0.06 | 0.22 |
| ENSRNOG00000035085 | *SNORA73* | 3.90 | 13.19 | 3.38 |
| ENSRNOG00000035170 | *U1* | 0.17 | 0.38 | 2.18 |
| ENSRNOG00000035191 | *U6* | 3.24 | 8.67 | 2.68 |
| ENSRNOG00000035252 | *U6* | 0.20 | 0.46 | 2.34 |
| ENSRNOG00000035273 | *U1* | 0.17 | 0.38 | 2.18 |
| ENSRNOG00000035274 | *U6* | 0.11 | 2.18 | 18.97 |
| ENSRNOG00000035375 | *U6* | 0.05 | 0.27 | 5.24 |
| ENSRNOG00000035410 | *U1* | 0.17 | 0.38 | 2.18 |
| ENSRNOG00000035702 | *U6* | 0.10 | 0.49 | 5.04 |
| ENSRNOG00000035783 | *SNORA32* | 0.06 | 3.24 | 55.78 |
| ENSRNOG00000035824 | *U1* | 0.17 | 0.38 | 2.18 |
| ENSRNOG00000035978 | *SNORA32* | 0.27 | 2.29 | 8.32 |
| ENSRNOG00000036221 | *U6* | 0.09 | 0.46 | 5.39 |
| ENSRNOG00000036451 | *-* | 0.06 | 0.18 | 2.92 |
| ENSRNOG00000036505 | *U1* | 0.17 | 0.38 | 2.18 |
| ENSRNOG00000036506 | *U1* | 0.17 | 0.38 | 2.18 |
| ENSRNOG00000036673 | *Sectm1b* | 5.10 | 0.45 | 0.09 |
| ENSRNOG00000036862 | *F1M9U6_RAT* | 9.38 | 4.13 | 0.44 |
| ENSRNOG00000036983 | *F1M848_RAT* | 0.12 | 0.40 | 3.41 |
| ENSRNOG00000037118 | *LOC100363228* | 4.90 | 2.14 | 0.44 |
| ENSRNOG00000037165 | *D4A8W9_RAT* | 2.90 | 0.48 | 0.17 |
| ENSRNOG00000037364 | *LOC100362023* | 6.27 | 3.06 | 0.49 |
| ENSRNOG00000037374 | *D3ZPQ1_RAT* | 0.14 | 0.38 | 2.73 |
| ENSRNOG00000037514 | *Qser1* | 4.82 | 2.35 | 0.49 |
| ENSRNOG00000037715 | *F1M046_RAT* | 4.51 | 2.03 | 0.45 |
| ENSRNOG00000037753 | *Slc10a2* | 7.00 | 2.51 | 0.36 |
| ENSRNOG00000037765 | *Lims1* | 5.83 | 2.32 | 0.40 |
| ENSRNOG00000038933 | *D3ZF12_RAT* | 9.23 | 3.23 | 0.35 |
| ENSRNOG00000038989 | *D3ZSD6_RAT* | 28.69 | 2.23 | 0.08 |
| ENSRNOG00000039063 | *LOC681355* | 6.20 | 2.09 | 0.34 |
| ENSRNOG00000039630 | *LOC290577* | 7.41 | 2.90 | 0.39 |
| ENSRNOG00000039717 | *Ipo11* | 9.58 | 2.55 | 0.27 |
| ENSRNOG00000040195 | *F1LZT0_RAT* | 7.00 | 0.46 | 0.07 |
| ENSRNOG00000040537 | *SNORA40* | 2.00 | 0.29 | 0.15 |
| ENSRNOG00000040690 | *SNORD86* | 2.19 | 5.08 | 2.31 |
| ENSRNOG00000040790 | *SNORA40* | 2.00 | 0.29 | 0.15 |
| ENSRNOG00000041195 | *-* | 2.95 | 26.75 | 9.06 |
| ENSRNOG00000041281 | *-* | 0.39 | 0.02 | 0.06 |
| ENSRNOG00000041291 | *-* | 2.20 | 0.39 | 0.18 |
| ENSRNOG00000041296 | *-* | 3.44 | 86.55 | 25.17 |
| ENSRNOG00000041309 | *-* | 0.48 | 27.16 | 56.60 |
| ENSRNOG00000041330 | *-* | 8.06 | 19.51 | 2.42 |
| ENSRNOG00000041331 | *-* | 0.05 | 2.31 | 47.15 |
| ENSRNOG00000041357 | *-* | 0.14 | 3.50 | 25.00 |
| ENSRNOG00000041386 | *-* | 3.61 | 23.12 | 6.40 |
| ENSRNOG00000041387 | *-* | 0.11 | 2.63 | 23.47 |
| ENSRNOG00000041406 | *-* | 0.28 | 2.17 | 7.71 |
| ENSRNOG00000041412 | *-* | 2.24 | 0.26 | 0.12 |
| ENSRNOG00000041459 | *-* | 0.12 | 0.36 | 2.97 |
| ENSRNOG00000041622 | *-* | 0.01 | 0.02 | 2.24 |
| ENSRNOG00000041624 | *-* | 12.91 | 2.32 | 0.18 |
| ENSRNOG00000041967 | *SNORA2* | 2.63 | 15.77 | 6.00 |
| ENSRNOG00000042072 | *Arhgdig* | 0.41 | 2.02 | 4.93 |
| ENSRNOG00000042174 | *F1M9D9_RAT* | 4.70 | 2.08 | 0.44 |
| ENSRNOG00000042244 | *F1LY42_RAT* | 2.09 | 0.05 | 0.02 |
| ENSRNOG00000042395 | *F1M058_RAT* | 7.61 | 2.15 | 0.28 |
| ENSRNOG00000042537 | *Q6LCG2_RAT* | 6.12 | 2.73 | 0.45 |
| ENSRNOG00000042540 | *Mef2a* | 8.45 | 2.05 | 0.24 |
| ENSRNOG00000042565 | *-* | 8.55 | 2.08 | 0.24 |
| ENSRNOG00000042580 | *F1LU95_RAT* | 2.14 | 0.46 | 0.22 |
| ENSRNOG00000042767 | *F1M6P7_RAT* | 2.74 | 0.34 | 0.13 |
| ENSRNOG00000042909 | *F1LZX4_RAT* | 0.11 | 0.50 | 4.67 |
| ENSRNOG00000043003 | *LOC689939* | 7.24 | 2.06 | 0.28 |
| ENSRNOG00000043006 | *Epm2aip1* | 5.49 | 2.34 | 0.43 |
| ENSRNOG00000043123 | *Rnf128* | 2.03 | 0.50 | 0.25 |
| ENSRNOG00000043352 | *D3ZVJ1_RAT* | 0.41 | 2.48 | 6.01 |
| ENSRNOG00000043560 | *-* | 27.43 | 2.65 | 0.10 |
| ENSRNOG00000043844 | *-* | 0.01 | 0.04 | 3.35 |
| ENSRNOG00000043936 | *-* | 5.80 | 0.36 | 0.06 |
| ENSRNOG00000044290 | *SNORA25* | 0.25 | 2.66 | 10.43 |
| ENSRNOG00000045194 | *SNORA44* | 0.04 | 0.10 | 2.23 |
| ENSRNOG00000045332 | *7SK* | 0.14 | 0.04 | 0.30 |

# Supplementary Table 3

**Table S3 Significantly regulated KEGG pathways in kidney of prenatal dexamethasone (DEX) exposure, melatonin, and DEX+melatonin offspring vs. control at 1 week of age**

| [**Term**](http://david.abcc.ncifcrf.gov/chartReport.jsp?d-16544-s=2&d-16544-o=2&d-16544-p=1&annot=47) | [**Count**](http://david.abcc.ncifcrf.gov/chartReport.jsp?d-16544-s=5&d-16544-o=1&d-16544-p=1&annot=47) | [**%**](http://david.abcc.ncifcrf.gov/chartReport.jsp?d-16544-s=6&d-16544-o=1&d-16544-p=1&annot=47) | [**P-Value**](http://david.abcc.ncifcrf.gov/chartReport.jsp?d-16544-s=7&d-16544-o=1&d-16544-p=1&annot=47) | [**Benjamini**](http://david.abcc.ncifcrf.gov/chartReport.jsp?d-16544-s=8&d-16544-o=1&d-16544-p=1&annot=47) |
| --- | --- | --- | --- | --- |
| **DEX: 12** |  |  |  |  |
| [Systemic lupus erythematosus](http://david.abcc.ncifcrf.gov/kegg.jsp?path=rno05322$Systemic%20lupus%20erythematosus&termId=470065501&source=kegg) | 13 | 1.1 | 5.0E-4 | 7.4E-2 |
| [Chemokine signaling pathway](http://david.abcc.ncifcrf.gov/kegg.jsp?path=rno04062$Chemokine%20signaling%20pathway&termId=470065419&source=kegg) | 15 | 1.2 | 1.7E-2 | 7.4E-1 |
| [Axon guidance](http://david.abcc.ncifcrf.gov/kegg.jsp?path=rno04360$Axon%20guidance&termId=470065439&source=kegg) | 12 | 1.0 | 2.3E-2 | 6.9E-1 |
| [Hedgehog signaling pathway](http://david.abcc.ncifcrf.gov/kegg.jsp?path=rno04340$Hedgehog%20signaling%20pathway&termId=470065437&source=kegg) | 7 | 0.6 | 2.5E-2 | 6.2E-1 |
| [Progesterone-mediated oocyte maturation](http://david.abcc.ncifcrf.gov/kegg.jsp?path=rno04914$Progesterone-mediated%20oocyte%20maturation&termId=470065472&source=kegg) | 9 | 0.7 | 3.6E-2 | 6.8E-1 |
| [Cell cycle](http://david.abcc.ncifcrf.gov/kegg.jsp?path=rno04110$Cell%20cycle&termId=470065422&source=kegg) | 11 | 0.9 | 4.9E-2 | 7.2E-1 |
| [ECM-receptor interaction](http://david.abcc.ncifcrf.gov/kegg.jsp?path=rno04512$ECM-receptor%20interaction&termId=470065442&source=kegg) | 8 | 0.6 | 6.3E-2 | 7.6E-1 |
| [Basal cell carcinoma](http://david.abcc.ncifcrf.gov/kegg.jsp?path=rno05217$Basal%20cell%20carcinoma&termId=470065492&source=kegg) | 6 | 0.5 | 7.1E-2 | 7.6E-1 |
| [Focal adhesion](http://david.abcc.ncifcrf.gov/kegg.jsp?path=rno04510$Focal%20adhesion&termId=470065441&source=kegg) | 14 | 1.1 | 8.5E-2 | 7.8E-1 |
| [Biosynthesis of unsaturated fatty acids](http://david.abcc.ncifcrf.gov/kegg.jsp?path=rno01040$Biosynthesis%20of%20unsaturated%20fatty%20acids&termId=470065399&source=kegg) | 4 | 0.3 | 8.5E-2 | 7.5E-1 |
| [PPAR signaling pathway](http://david.abcc.ncifcrf.gov/kegg.jsp?path=rno03320$PPAR%20signaling%20pathway&termId=470065409&source=kegg) | 7 | 0.6 | 9.0E-2 | 7.3E-1 |
| [Antigen processing and presentation](http://david.abcc.ncifcrf.gov/kegg.jsp?path=rno04612$Antigen%20processing%20and%20presentation&termId=470065448&source=kegg) | 8 | 0.6 | 9.0E-2 | 7.0E-1 |
| **Melatonin:55** |  |  |  |  |
| [Focal adhesion](http://david.abcc.ncifcrf.gov/kegg.jsp?path=rno04510$Focal%20adhesion&termId=470065441&source=kegg) | 52 | 1.8 | 3.5E-7 | 6.6E-5 |
| [Adherens junction](http://david.abcc.ncifcrf.gov/kegg.jsp?path=rno04520$Adherens%20junction&termId=470065444&source=kegg) | 26 | 0.9 | 3.0E-6 | 2.8E-4 |
| [Axon guidance](http://david.abcc.ncifcrf.gov/kegg.jsp?path=rno04360$Axon%20guidance&termId=470065439&source=kegg) | 36 | 1.2 | 7.2E-6 | 4.5E-4 |
| [Regulation of actin cytoskeleton](http://david.abcc.ncifcrf.gov/kegg.jsp?path=rno04810$Regulation%20of%20actin%20cytoskeleton&termId=470065469&source=kegg) | 51 | 1.7 | 7.6E-6 | 3.6E-4 |
| [Pathways in cancer](http://david.abcc.ncifcrf.gov/kegg.jsp?path=rno05200$Pathways%20in%20cancer&termId=470065484&source=kegg) | 66 | 2.2 | 7.3E-5 | 2.8E-3 |
| [Prostate cancer](http://david.abcc.ncifcrf.gov/kegg.jsp?path=rno05215$Prostate%20cancer&termId=470065490&source=kegg) | 26 | 0.9 | 1.3E-4 | 4.0E-3 |
| [ErbB signaling pathway](http://david.abcc.ncifcrf.gov/kegg.jsp?path=rno04012$ErbB%20signaling%20pathway&termId=470065416&source=kegg) | 25 | 0.9 | 1.3E-4 | 3.5E-3 |
| [Chemokine signaling pathway](http://david.abcc.ncifcrf.gov/kegg.jsp?path=rno04062$Chemokine%20signaling%20pathway&termId=470065419&source=kegg) | 40 | 1.4 | 2.3E-4 | 5.4E-3 |
| [Insulin signaling pathway](http://david.abcc.ncifcrf.gov/kegg.jsp?path=rno04910$Insulin%20signaling%20pathway&termId=470065470&source=kegg) | 33 | 1.1 | 2.6E-4 | 5.4E-3 |
| [Chronic myeloid leukemia](http://david.abcc.ncifcrf.gov/kegg.jsp?path=rno05220$Chronic%20myeloid%20leukemia&termId=470065495&source=kegg) | 22 | 0.7 | 3.8E-4 | 7.2E-3 |
| [Tryptophan metabolism](http://david.abcc.ncifcrf.gov/kegg.jsp?path=rno00380$Tryptophan%20metabolism&termId=470065344&source=kegg) | 15 | 0.5 | 7.0E-4 | 1.2E-2 |
| [mTOR signaling pathway](http://david.abcc.ncifcrf.gov/kegg.jsp?path=rno04150$mTOR%20signaling%20pathway&termId=470065430&source=kegg) | 17 | 0.6 | 7.6E-4 | 1.2E-2 |
| [Renal cell carcinoma](http://david.abcc.ncifcrf.gov/kegg.jsp?path=rno05211$Renal%20cell%20carcinoma&termId=470065486&source=kegg) | 20 | 0.7 | 9.0E-4 | 1.3E-2 |
| [Melanoma](http://david.abcc.ncifcrf.gov/kegg.jsp?path=rno05218$Melanoma&termId=470065493&source=kegg) | 20 | 0.7 | 9.0E-4 | 1.3E-2 |
| [PPAR signaling pathway](http://david.abcc.ncifcrf.gov/kegg.jsp?path=rno03320$PPAR%20signaling%20pathway&termId=470065409&source=kegg) | 20 | 0.7 | 1.3E-3 | 1.8E-2 |
| [Butanoate metabolism](http://david.abcc.ncifcrf.gov/kegg.jsp?path=rno00650$Butanoate%20metabolism&termId=470065378&source=kegg) | 12 | 0.4 | 2.1E-3 | 2.6E-2 |
| [Pancreatic cancer](http://david.abcc.ncifcrf.gov/kegg.jsp?path=rno05212$Pancreatic%20cancer&termId=470065487&source=kegg) | 19 | 0.6 | 2.4E-3 | 2.8E-2 |
| [Colorectal cancer](http://david.abcc.ncifcrf.gov/kegg.jsp?path=rno05210$Colorectal%20cancer&termId=470065485&source=kegg) | 21 | 0.7 | 2.8E-3 | 3.1E-2 |
| [Endometrial cancer](http://david.abcc.ncifcrf.gov/kegg.jsp?path=rno05213$Endometrial%20cancer&termId=470065488&source=kegg) | 15 | 0.5 | 4.3E-3 | 4.4E-2 |
| [Endocytosis](http://david.abcc.ncifcrf.gov/kegg.jsp?path=rno04144$Endocytosis&termId=470065429&source=kegg) | 40 | 1.4 | 5.6E-3 | 5.4E-2 |
| [MAPK signaling pathway](http://david.abcc.ncifcrf.gov/kegg.jsp?path=rno04010$MAPK%20signaling%20pathway&termId=470065415&source=kegg) | 50 | 1.7 | 5.9E-3 | 5.5E-2 |
| [Melanogenesis](http://david.abcc.ncifcrf.gov/kegg.jsp?path=rno04916$Melanogenesis&termId=470065473&source=kegg) | 22 | 0.7 | 6.1E-3 | 5.3E-2 |
| [Aldosterone-regulated sodium reabsorption](http://david.abcc.ncifcrf.gov/kegg.jsp?path=rno04960$Aldosterone-regulated%20sodium%20reabsorption&termId=470065478&source=kegg) | 13 | 0.4 | 6.9E-3 | 5.8E-2 |
| [Fc gamma R-mediated phagocytosis](http://david.abcc.ncifcrf.gov/kegg.jsp?path=rno04666$Fc%20gamma%20R-mediated%20phagocytosis&termId=470065460&source=kegg) | 21 | 0.7 | 7.7E-3 | 6.2E-2 |
| [Biosynthesis of unsaturated fatty acids](http://david.abcc.ncifcrf.gov/kegg.jsp?path=rno01040$Biosynthesis%20of%20unsaturated%20fatty%20acids&termId=470065399&source=kegg) | 9 | 0.3 | 8.4E-3 | 6.4E-2 |
| [Small cell lung cancer](http://david.abcc.ncifcrf.gov/kegg.jsp?path=rno05222$Small%20cell%20lung%20cancer&termId=470065497&source=kegg) | 20 | 0.7 | 8.6E-3 | 6.3E-2 |
| [Wnt signaling pathway](http://david.abcc.ncifcrf.gov/kegg.jsp?path=rno04310$Wnt%20signaling%20pathway&termId=470065434&source=kegg) | 30 | 1.0 | 1.0E-2 | 7.3E-2 |
| [Basal cell carcinoma](http://david.abcc.ncifcrf.gov/kegg.jsp?path=rno05217$Basal%20cell%20carcinoma&termId=470065492&source=kegg) | 14 | 0.5 | 1.1E-2 | 7.6E-2 |
| [T cell receptor signaling pathway](http://david.abcc.ncifcrf.gov/kegg.jsp?path=rno04660$T%20cell%20receptor%20signaling%20pathway&termId=470065457&source=kegg) | 24 | 0.8 | 1.1E-2 | 7.4E-2 |
| [Non-small cell lung cancer](http://david.abcc.ncifcrf.gov/kegg.jsp?path=rno05223$Non-small%20cell%20lung%20cancer&termId=470065498&source=kegg) | 14 | 0.5 | 1.3E-2 | 8.3E-2 |
| [Tight junction](http://david.abcc.ncifcrf.gov/kegg.jsp?path=rno04530$Tight%20junction&termId=470065445&source=kegg) | 27 | 0.9 | 1.5E-2 | 8.9E-2 |
| [Vascular smooth muscle contraction](http://david.abcc.ncifcrf.gov/kegg.jsp?path=rno04270$Vascular%20smooth%20muscle%20contraction&termId=470065433&source=kegg) | 24 | 0.8 | 1.7E-2 | 1.0E-1 |
| [beta-Alanine metabolism](http://david.abcc.ncifcrf.gov/kegg.jsp?path=rno00410$beta-Alanine%20metabolism&termId=470065346&source=kegg) | 8 | 0.3 | 1.8E-2 | 1.0E-1 |
| [Neurotrophin signaling pathway](http://david.abcc.ncifcrf.gov/kegg.jsp?path=rno04722$Neurotrophin%20signaling%20pathway&termId=470065465&source=kegg) | 26 | 0.9 | 1.8E-2 | 1.0E-1 |
| [Jak-STAT signaling pathway](http://david.abcc.ncifcrf.gov/kegg.jsp?path=rno04630$Jak-STAT%20signaling%20pathway&termId=470065454&source=kegg) | 28 | 1.0 | 1.9E-2 | 1.0E-1 |
| [Glioma](http://david.abcc.ncifcrf.gov/kegg.jsp?path=rno05214$Glioma&termId=470065489&source=kegg) | 15 | 0.5 | 2.2E-2 | 1.1E-1 |
| [Adipocytokine signaling pathway](http://david.abcc.ncifcrf.gov/kegg.jsp?path=rno04920$Adipocytokine%20signaling%20pathway&termId=470065474&source=kegg) | 16 | 0.5 | 2.2E-2 | 1.1E-1 |
| [Apoptosis](http://david.abcc.ncifcrf.gov/kegg.jsp?path=rno04210$Apoptosis&termId=470065431&source=kegg) | 19 | 0.6 | 2.3E-2 | 1.1E-1 |
| [Glutathione metabolism](http://david.abcc.ncifcrf.gov/kegg.jsp?path=rno00480$Glutathione%20metabolism&termId=470065352&source=kegg) | 13 | 0.4 | 2.3E-2 | 1.1E-1 |
| [Valine, leucine and isoleucine degradation](http://david.abcc.ncifcrf.gov/kegg.jsp?path=rno00280$Valine,%20leucine%20and%20isoleucine%20degradation&termId=470065337&source=kegg) | 12 | 0.4 | 3.0E-2 | 1.4E-1 |
| [Lysine degradation](http://david.abcc.ncifcrf.gov/kegg.jsp?path=rno00310$Lysine%20degradation&termId=470065339&source=kegg) | 11 | 0.4 | 3.3E-2 | 1.5E-1 |
| [Renin-angiotensin system](http://david.abcc.ncifcrf.gov/kegg.jsp?path=rno04614$Renin-angiotensin%20system&termId=470065449&source=kegg) | 7 | 0.2 | 3.6E-2 | 1.6E-1 |
| [Fatty acid metabolism](http://david.abcc.ncifcrf.gov/kegg.jsp?path=rno00071$Fatty%20acid%20metabolism&termId=470065324&source=kegg) | 11 | 0.4 | 3.9E-2 | 1.6E-1 |
| [Limonene and pinene degradation](http://david.abcc.ncifcrf.gov/kegg.jsp?path=rno00903$Limonene%20and%20pinene%20degradation&termId=470065392&source=kegg) | 5 | 0.2 | 4.4E-2 | 1.8E-1 |
| [Type II diabetes mellitus](http://david.abcc.ncifcrf.gov/kegg.jsp?path=rno04930$Type%20II%20diabetes%20mellitus&termId=470065475&source=kegg) | 12 | 0.4 | 4.6E-2 | 1.8E-1 |
| [Acute myeloid leukemia](http://david.abcc.ncifcrf.gov/kegg.jsp?path=rno05221$Acute%20myeloid%20leukemia&termId=470065496&source=kegg) | 13 | 0.4 | 4.6E-2 | 1.8E-1 |
| [Progesterone-mediated oocyte maturation](http://david.abcc.ncifcrf.gov/kegg.jsp?path=rno04914$Progesterone-mediated%20oocyte%20maturation&termId=470065472&source=kegg) | 18 | 0.6 | 5.2E-2 | 2.0E-1 |
| [Ubiquitin mediated proteolysis](http://david.abcc.ncifcrf.gov/kegg.jsp?path=rno04120$Ubiquitin%20mediated%20proteolysis&termId=470065425&source=kegg) | 24 | 0.8 | 5.4E-2 | 2.0E-1 |
| [B cell receptor signaling pathway](http://david.abcc.ncifcrf.gov/kegg.jsp?path=rno04662$B%20cell%20receptor%20signaling%20pathway&termId=470065458&source=kegg) | 16 | 0.5 | 5.5E-2 | 2.0E-1 |
| [Leukocyte transendothelial migration](http://david.abcc.ncifcrf.gov/kegg.jsp?path=rno04670$Leukocyte%20transendothelial%20migration&termId=470065461&source=kegg) | 22 | 0.7 | 6.3E-2 | 2.2E-1 |
| [Hedgehog signaling pathway](http://david.abcc.ncifcrf.gov/kegg.jsp?path=rno04340$Hedgehog%20signaling%20pathway&termId=470065437&source=kegg) | 12 | 0.4 | 6.6E-2 | 2.3E-1 |
| [Toll-like receptor signaling pathway](http://david.abcc.ncifcrf.gov/kegg.jsp?path=rno04620$Toll-like%20receptor%20signaling%20pathway&termId=470065450&source=kegg) | 18 | 0.6 | 6.9E-2 | 2.3E-1 |
| [Prion diseases](http://david.abcc.ncifcrf.gov/kegg.jsp?path=rno05020$Prion%20diseases&termId=470065483&source=kegg) | 9 | 0.3 | 7.5E-2 | 2.5E-1 |
| [Thyroid cancer](http://david.abcc.ncifcrf.gov/kegg.jsp?path=rno05216$Thyroid%20cancer&termId=470065491&source=kegg) | 8 | 0.3 | 8.4E-2 | 2.7E-1 |
| [Alanine, aspartate and glutamate metabolism](http://david.abcc.ncifcrf.gov/kegg.jsp?path=rno00250$Alanine,%20aspartate%20and%20glutamate%20metabolism&termId=470065334&source=kegg) | 8 | 0.3 | 8.4E-2 | 2.7E-1 |
| **DEX+Melatonin: 36** |  |  |  |  |
| [PPAR signaling pathway](http://david.abcc.ncifcrf.gov/kegg.jsp?path=rno03320$PPAR%20signaling%20pathway&termId=470065409&source=kegg) | 25 | 1.1 | 1.6E-7 | 2.9E-5 |
| [Renin-angiotensin system](http://david.abcc.ncifcrf.gov/kegg.jsp?path=rno04614$Renin-angiotensin%20system&termId=470065449&source=kegg) | 10 | 0.4 | 1.1E-4 | 9.7E-3 |
| [Focal adhesion](http://david.abcc.ncifcrf.gov/kegg.jsp?path=rno04510$Focal%20adhesion&termId=470065441&source=kegg) | 36 | 1.6 | 1.6E-3 | 9.2E-2 |
| [Nitrogen metabolism](http://david.abcc.ncifcrf.gov/kegg.jsp?path=rno00910$Nitrogen%20metabolism&termId=470065393&source=kegg) | 9 | 0.4 | 2.0E-3 | 8.7E-2 |
| [Fatty acid metabolism](http://david.abcc.ncifcrf.gov/kegg.jsp?path=rno00071$Fatty%20acid%20metabolism&termId=470065324&source=kegg) | 12 | 0.5 | 4.0E-3 | 1.4E-1 |
| [Axon guidance](http://david.abcc.ncifcrf.gov/kegg.jsp?path=rno04360$Axon%20guidance&termId=470065439&source=kegg) | 25 | 1.1 | 4.2E-3 | 1.2E-1 |
| [Tryptophan metabolism](http://david.abcc.ncifcrf.gov/kegg.jsp?path=rno00380$Tryptophan%20metabolism&termId=470065344&source=kegg) | 12 | 0.5 | 4.9E-3 | 1.2E-1 |
| [Glycine, serine and threonine metabolism](http://david.abcc.ncifcrf.gov/kegg.jsp?path=rno00260$Glycine,%20serine%20and%20threonine%20metabolism&termId=470065335&source=kegg) | 10 | 0.4 | 5.4E-3 | 1.2E-1 |
| [Glutathione metabolism](http://david.abcc.ncifcrf.gov/kegg.jsp?path=rno00480$Glutathione%20metabolism&termId=470065352&source=kegg) | 13 | 0.6 | 5.8E-3 | 1.1E-1 |
| [Regulation of actin cytoskeleton](http://david.abcc.ncifcrf.gov/kegg.jsp?path=rno04810$Regulation%20of%20actin%20cytoskeleton&termId=470065469&source=kegg) | 35 | 1.6 | 9.0E-3 | 1.5E-1 |
| [Pantothenate and CoA biosynthesis](http://david.abcc.ncifcrf.gov/kegg.jsp?path=rno00770$Pantothenate%20and%20CoA%20biosynthesis&termId=470065385&source=kegg) | 6 | 0.3 | 1.7E-2 | 2.5E-1 |
| [Vascular smooth muscle contraction](http://david.abcc.ncifcrf.gov/kegg.jsp?path=rno04270$Vascular%20smooth%20muscle%20contraction&termId=470065433&source=kegg) | 21 | 0.9 | 1.7E-2 | 2.3E-1 |
| [ErbB signaling pathway](http://david.abcc.ncifcrf.gov/kegg.jsp?path=rno04012$ErbB%20signaling%20pathway&termId=470065416&source=kegg) | 17 | 0.8 | 1.8E-2 | 2.3E-1 |
| [Pathways in cancer](http://david.abcc.ncifcrf.gov/kegg.jsp?path=rno05200$Pathways%20in%20cancer&termId=470065484&source=kegg) | 47 | 2.1 | 2.1E-2 | 2.4E-1 |
| [Propanoate metabolism](http://david.abcc.ncifcrf.gov/kegg.jsp?path=rno00640$Propanoate%20metabolism&termId=470065377&source=kegg) | 9 | 0.4 | 2.1E-2 | 2.3E-1 |
| [Endocytosis](http://david.abcc.ncifcrf.gov/kegg.jsp?path=rno04144$Endocytosis&termId=470065429&source=kegg) | 32 | 1.4 | 2.5E-2 | 2.5E-1 |
| [Adipocytokine signaling pathway](http://david.abcc.ncifcrf.gov/kegg.jsp?path=rno04920$Adipocytokine%20signaling%20pathway&termId=470065474&source=kegg) | 14 | 0.6 | 2.5E-2 | 2.4E-1 |
| [Systemic lupus erythematosus](http://david.abcc.ncifcrf.gov/kegg.jsp?path=rno05322$Systemic%20lupus%20erythematosus&termId=470065501&source=kegg) | 17 | 0.8 | 3.0E-2 | 2.7E-1 |
| [Small cell lung cancer](http://david.abcc.ncifcrf.gov/kegg.jsp?path=rno05222$Small%20cell%20lung%20cancer&termId=470065497&source=kegg) | 16 | 0.7 | 3.1E-2 | 2.6E-1 |
| [Aldosterone-regulated sodium reabsorption](http://david.abcc.ncifcrf.gov/kegg.jsp?path=rno04960$Aldosterone-regulated%20sodium%20reabsorption&termId=470065478&source=kegg) | 10 | 0.4 | 3.7E-2 | 2.9E-1 |
| [Hematopoietic cell lineage](http://david.abcc.ncifcrf.gov/kegg.jsp?path=rno04640$Hematopoietic%20cell%20lineage&termId=470065455&source=kegg) | 15 | 0.7 | 3.8E-2 | 2.9E-1 |
| [Drug metabolism](http://david.abcc.ncifcrf.gov/kegg.jsp?path=rno00982$Drug%20metabolism&termId=470065397&source=kegg) | 14 | 0.6 | 4.3E-2 | 3.1E-1 |
| [Hedgehog signaling pathway](http://david.abcc.ncifcrf.gov/kegg.jsp?path=rno04340$Hedgehog%20signaling%20pathway&termId=470065437&source=kegg) | 11 | 0.5 | 4.9E-2 | 3.3E-1 |
| [ECM-receptor interaction](http://david.abcc.ncifcrf.gov/kegg.jsp?path=rno04512$ECM-receptor%20interaction&termId=470065442&source=kegg) | 15 | 0.7 | 5.0E-2 | 3.3E-1 |
| [Metabolism of xenobiotics by cytochrome P450](http://david.abcc.ncifcrf.gov/kegg.jsp?path=rno00980$Metabolism%20of%20xenobiotics%20by%20cytochrome%20P450&termId=470065396&source=kegg) | 12 | 0.5 | 5.4E-2 | 3.4E-1 |
| [Butanoate metabolism](http://david.abcc.ncifcrf.gov/kegg.jsp?path=rno00650$Butanoate%20metabolism&termId=470065378&source=kegg) | 8 | 0.4 | 5.9E-2 | 3.5E-1 |
| [Renal cell carcinoma](http://david.abcc.ncifcrf.gov/kegg.jsp?path=rno05211$Renal%20cell%20carcinoma&termId=470065486&source=kegg) | 13 | 0.6 | 6.4E-2 | 3.6E-1 |
| [Chemokine signaling pathway](http://david.abcc.ncifcrf.gov/kegg.jsp?path=rno04062$Chemokine%20signaling%20pathway&termId=470065419&source=kegg) | 26 | 1.2 | 7.0E-2 | 3.8E-1 |
| [Apoptosis](http://david.abcc.ncifcrf.gov/kegg.jsp?path=rno04210$Apoptosis&termId=470065431&source=kegg) | 15 | 0.7 | 7.0E-2 | 3.7E-1 |
| [Insulin signaling pathway](http://david.abcc.ncifcrf.gov/kegg.jsp?path=rno04910$Insulin%20signaling%20pathway&termId=470065470&source=kegg) | 21 | 0.9 | 7.3E-2 | 3.7E-1 |
| [Cell adhesion molecules (CAMs)](http://david.abcc.ncifcrf.gov/kegg.jsp?path=rno04514$Cell%20adhesion%20molecules%20(CAMs)&termId=470065443&source=kegg) | 23 | 1.0 | 7.4E-2 | 3.7E-1 |
| [Lysosome](http://david.abcc.ncifcrf.gov/kegg.jsp?path=rno04142$Lysosome&termId=470065428&source=kegg) | 19 | 0.8 | 7.7E-2 | 3.7E-1 |
| [beta-Alanine metabolism](http://david.abcc.ncifcrf.gov/kegg.jsp?path=rno00410$beta-Alanine%20metabolism&termId=470065346&source=kegg) | 6 | 0.3 | 8.1E-2 | 3.8E-1 |
| [O-Glycan biosynthesis](http://david.abcc.ncifcrf.gov/kegg.jsp?path=rno00512$O-Glycan%20biosynthesis&termId=470065356&source=kegg) | 7 | 0.3 | 8.6E-2 | 3.8E-1 |
| [Colorectal cancer](http://david.abcc.ncifcrf.gov/kegg.jsp?path=rno05210$Colorectal%20cancer&termId=470065485&source=kegg) | 14 | 0.6 | 9.3E-2 | 4.0E-1 |
| [Adherens junction](http://david.abcc.ncifcrf.gov/kegg.jsp?path=rno04520$Adherens%20junction&termId=470065444&source=kegg) | 13 | 0.6 | 9.8E-2 | 4.1E-1 |
